# EVOO Promotes a Less Atherogenic Profile Than Sunflower Oil in Smooth Muscle Cells Through the Extracellular Vesicles Secreted by Endothelial Cells

**DOI:** 10.3389/fnut.2022.867745

**Published:** 2022-04-12

**Authors:** Concepción Santiago-Fernandez, Cristina Rodríguez-Díaz, Ailec Ho-Plagaro, Carolina Gutierrez-Repiso, Wilfredo Oliva-Olivera, Flores Martin-Reyes, Virginia Mela, Rocío Bautista, Mónicas Tome, Josefa Gómez-Maldonado, Francisco J. Tinahones, Eduardo Garcia-Fuentes, Lourdes Garrido-Sánchez

**Affiliations:** ^1^Instituto de Investigación Biomédica de Málaga-IBIMA, Málaga, Spain; ^2^Unidad de Gestión Clínica de Aparato Digestivo, Hospital Universitario Virgen de la Victoria, Málaga, Spain; ^3^Unidad de Gestión Clínica de Endocrinología y Nutrición, Hospital Universitario Virgen de la Victoria, Málaga, Spain; ^4^Centro de Investigación Biomédica en Red-Fisiopatología de la Obesidad y Nutrición (CIBEROBN), Instituto de Salud Carlos III, Málaga, Spain; ^5^Facultad de Medicina, Universidad de Málaga, Málaga, Spain; ^6^Plataforma Andaluza de Bioinformática-Supercomputing and Bioinnovation Center, Universidad de Málaga, Málaga, Spain; ^7^Unidad de Gestión Clínica de Endocrinología y Nutrición, Hospital Regional Universitario, Málaga, Spain; ^8^Unidad de Genómica y Ultrasecuenciación-Supercomputing and Bioinnovation Center, Universidad de Málaga, Málaga, Spain; ^9^Centro de Investigación Biomédica en Red de Enfermedades Hepáticas y Digestivas (CIBEREHD), Instituto de Salud Carlos III, Málaga, Spain

**Keywords:** miRNAs, HUVECs, extra-virgin olive oil, sunflower oil, smooth muscle cells (SMCs), extracellular vesicles (EVs)

## Abstract

**Background:**

Little is known about the effect of extra virgin olive (EVOO) and sunflower oil (SO) on the composition of extracellular vesicles (EVs) secreted by endothelial cells and the effects of these EVs on smooth muscle cells (SMCs). These cells play an important role in the development of atherosclerosis.

**Methods:**

We evaluated the effects of endothelial cells-derived EVs incubated with triglyceride-rich lipoproteins obtained after a high-fat meal with EVOO (EVOO-EVs) and SO (SO-EVs), on the transcriptomic profile of SMCs.

**Results:**

We found 41 upregulated and 19 downregulated differentially expressed (DE)-miRNAs in EVOO-EVs. Afterwards, SMCs were incubated with EVOO-EVs and SO-EVs. SMCs incubated with SO-EVs showed a greater number of DE-mRNA involved in pathways related to cancer, focal adhesion, regulation of actin cytoskeleton, and MAPK, toll-like receptor, chemokine and Wnt signaling pathways than in SMCs incubated with EVOO-EVs. These DE-mRNAs were involved in biological processes related to the response to endogenous stimulus, cell motility, regulation of intracellular signal transduction and cell population proliferation.

**Conclusion:**

EVOO and SO can differently modify the miRNA composition of HUVEC-derived EVs. These EVs can regulate the SMCs transcriptomic profile, with SO-EVs promoting a profile more closely linked to the development of atherosclerosis than EVOO-EVs.

## Introduction

Epidemiological studies have shown an inverse relationship between the intake of a Mediterranean diet and cardiovascular disease (1). Numerous studies have evaluated the role of Mediterranean diet on different variables related to cardiovascular and confirmed that it consumption was associated with a body weight loss, body composition remodeling, improvement of HDL quality and the prevention of HDL dysfunctionality, as well as a reduction of cardiovascular risk indexes (2, 3). However, the mechanisms of this association are not fully understood. For years, great importance has been given to oleic acid, the major fatty acid in extra-virgin olive oil (EVOO) and a fundamental constituent of the Mediterranean diet. This type of fatty acid has been associated with a decreased risk of developing cardiovascular disease (4). Also, this fatty acid prevents the increase of insulin resistance in myotubes (5, 6) and a reduction of inflammation and insulin resistance in smooth muscle cells (SMCs) *via* NF-κβ (5). More recently, importance has been given to the various minority components of EVOO, such as phenolic compounds, which are implicated in its heart-healthy properties (7, 8). EVOO is a fundamental ingredient of the Mediterranean diet and responsible for a large part of health benefits associated to this diet. Taken together, olive oil appears to have a direct heart-healthy effect.

The composition of triglycerides-rich lipoproteins (TRLs) is involved in the effects of different diets on the metabolism of endothelial cells (9), which are involved in the process related to cardiovascular diseases, such as atherosclerosis and endothelial dysfunction. The free fatty acids present in blood plasma, together the fatty acids liberated from TRLs by lipolisis, are transfered by different receptors, such as CD36 and other receptors, to endothelial cells. Also, these TRLs can non-specifically interact with the endothelial cells surface and be internalized by macro-pinocytosis after association with cell surface proteoglycans. This mechanism could be partly responsible for the effects produced by the minor components present in TRLs derived from the diet. However, these fatty acids also could enter the subendothelial space *via* a yet to be defined receptor, or *via* non-receptor movement either through or around the cells through channels created by the loosening of endothelial cells junctions (10).

In atherosclerosis and endothelial dysfunction, the interrelationship between endothelial and vascular SMCs is relevant since an alteration in this intercommunication can lead to the development of atherosclerosis (11). In this context, extracellular vesicles (EVs) could play an important role. EVs have an important role in cellular functions and processes such as angiogenesis, apoptosis, atherosclerosis, inflammation, coagulation, and tumoral dissemination among others (12). Previous studies have shown that endothelial cell-derived EVs could regulate the SMCs phenotype (13, 14) and could be involved in the development of atherosclerosis (15). Other studies have shown that endothelial cells can also modify the release of EVs and their content in response to different cardiovascular risk factors, such as TNFα and angiotensin II (14, 16, 17). However, little is known about the effects of different types of diets on the EVs composition of endothelial cell-derived EVs, and on the effects of these EVs on SMCs.

Given this lack of information, we conducted a study to evaluate the effects of EVs derived from endothelial cells incubated with the TRLs obtained after a high-fat (HF) meal with EVOO and sunflower oil (SO), on the gene expression of SMCs.

## Materials and Methods

### Design and Study Subjects

The subjects included were non-obese [body mass index (BMI) < 25.0 kg/m^2^] (*n* = 16) (Supplementary Table 1) (9). Two types of HF meal were administered: one type consisted of 50 gr of bread + 25 ml of EVOO to 8 subjects, and another type consisted of 50 gr of bread + 25 ml of SO to another 8 subjects. There are no significant differences between patients included in the group with EVOO and in the group with SO (Supplementary Table 1). Eight subjects (*n* = 4/group, 1 man and 3 women per group) were used to perform an ultrasequencing of miRNA libraries to analyse the miRNA expression pattern, and another 8 subjects more (*n* = 8 per group, total *n* = 16) were used for confirmatory analysis by RT-qPCR of the results obtained from sequencing. There are no significant differences between the group used for miRNA-Seq and that used for confirmatory analysis (data not shown). The subjects included in the study did not present any alteration in the carbohydrate or lipid metabolism, or medication against them, major cardiovascular disease in the 6 months prior to inclusion in the study, evidence of acute inflammatory disease or anti-inflammatory treatment, infectious disease, intake of drugs that may alter the lipid profile or metabolic parameters at the time of inclusion in the study, with a sports lifestyle. All subjects were of Caucasian origin. This study was carried out at the Virgen de la Victoria University Hospital (Malaga, Spain). Samples were processed and frozen immediately after their reception in the Regional University Hospital Biobank (Andalusian Public Health System Biobank). All participants gave their written informed consent and the study was reviewed and approved by the Malaga Provincial Research Ethics Committee (Malaga, Spain) (PI14/01306). A diagram of the study is shown in Supplementary Figure 1.

### Isolation of TRLs and Incubation of HUVECs

Isolation of TRLs was performed as previously described (9). Briefly, 3 ml of serum was obtained 3 h after HF meal intake and used for the isolation of the TRLs by density gradient ultracentrifugation at 50,000 × g for 60 min at 18°C. All the TRLs isolated were collected and immediately used for incubation of HUVECs [HUVECs (200P-05N), Sigma-Aldrich, St. Louis, MO] in M199 medium (Sigma-Aldrich, St. Louis, MO) supplemented with exosome-depleted FBS (20%) (Thermo Fisher Scientific Inc., Waltham, MA), human β-endothelial cell growth factor (5 mg/100 ml), glutamine (2 mM) and penicillin/streptomycin (1%). This incubation was maintained for 24 h (18), after which, culture medium was collected for EVs isolation.

### Isolation of EVs

EVs from HUVECs culture were isolated as described below to avoid the effect of other possible contaminants (e.g., cells debris, TRLs and most of proteins). The culture medium was centrifuged at 4,000 rpm, 10 min at 4°C and the supernatant was subjected to ultracentrifugation (Beckman XL-90 ultracentrifuge, Beckman Coulter) at 100,000 g for 90 min at 4°C without brake. The pellet with the EVs was subjected to a size-exclusion chromatography (SEC) using Izon qEVs 30 nm (Izon Science Europe Ltd, Oxford, UK), and the pooled fractions 7–9 (enriched in EVs) were concentrated by ultracentrifugation at 100,000 g for 90 min at 4°C. After, the EVs isolated were resuspended in 200 μl of M199 medium. Hundred microliter of EVs were used for the incubation of SMCs, and other aliquots were kept at −80°C for electron microscopy, western blot, nanoparticle tracking and miRNA analysis.

### Electron Microscopy of EVs

The isolated EVs (*n* = 2 per group) were fixed in 2% paraformaldehyde-−0.1 M PBS for 30 min. Glow discharge technique (60 s, 7.2 V, using a Bal-Tec MED 020 Coating System) was applied over carbon-coated copper grids, and immediately, these grids were placed on top of sample drops for 15 min. Then, the grids with adherent EVs were washed in a 0.1 M PBS drop and additional fixation in 1% glutaraldehyde was performed for 5 min. After washing properly in distilled water, the grids were contrasted with 1% uranyl acetate and embedded in methylcellulose. Excess fluid was removed and allowed to dry before examination with a transmission electron microscope FEI Tecnai G2 Spirit (ThermoFisher Scientific, Oregon, USA). All images were acquired using a Morada digital camera (Olympus Soft Image Solutions GmbH, Münster, Germany). The magnification used for the TEM images was 49,000 × .

### Nanoparticle Tracking Analysis

The EVs size and concentration were assessed using the NanoSight NS300 system (Malvern Panalytical, Malvern, UK) (*n* = 2). Particles were automatically tracked and sized-based on Brownian motion and the diffusion coefficient. The EVs were resuspended and diluted with 0.22 μm-filtered PBS at a concentration range of 10^9^ particles/mL, and 1 mL was used for NanoSight analysis. Five replicates of 30 s videos were captured to analyze concentration and size distribution of EVs at threshold detection of 5. Data analysis was performed using NanoSight analysis software.

### Western Blot of EVs

Protein concentration of EVs was determined with the BCA Protein Assay Reagent (Thermo Fisher Scientific Inc., Rockford, IL, USA). EVs (30 ug) (*n* = 2 per group) were subjected to SDS-PAGE on NB12-420 gels and electrotransferred on polyvinylidene fluoride membrane. Membranes were blocked in PBS-bovine serum albumin (BSA) 5% for 1 h. Membranes were incubated with a rabbit anti-CD9 antibody (ab92726) (Abcam, Cambridge, UK) overnight at 4°C. This protein was one of those that showed the best signal in previous studies in HUVECs cells (19, 20). Membranes were washed and incubated with horseradish peroxidase-conjugated secondary antibody [VeriBlot for IP Detection Reagent (HRP) (ab131366)] for 2 h at room temperature. The proteins were visualized by an ImageQuant LAS 4000 (GE Healthcare UK Limited, Buckinghamshire, England). Blots were analyzed using ImageJ software (NIH, Bethesda, MD).

### miRNA Extraction of EVs

Total RNA with an enhanced miRNA enrichment was extracted from EVs secreted by HUVEC with a miRNA specific kit (Maxwell^®^ 16 miRNA Tissue Kit, Promega) as previously described (9). An aliquot was used to perform an ultrasequencing of miRNA libraries to analyze the miRNA expression pattern (*n* = 4 per group) and another aliquot was used for confirmatory analysis by RT-qPCR (*n* = 8 per group) of the results obtained from sequencing. The cDNA was obtained with TaqMan™ Advanced miRNA cDNA Synthesis Kit (Thermo Fisher Scientific Inc, Waltham, MA). RNA purity was checked using the NanoVuePlus^®^ spectrophotometer (GE Healthcare Life Sciences, Pittsburgh, PA). The RNA concentration was measured using the Qubit^®^ RNA Assay Kit in the Qubit^®^ 3.0 Flurometer (Thermo Fisher Scientific Inc, Waltham, MA). Finally, RNA integrity was assessed using the RNA Nano 6000 Assay Kit with the Agilent Bioanalyzer 2100 system (Agilent Technologies, Santa Clara, CA).

### Library Preparation and Small RNA Sequencing

For sequencing, the small RNA transcripts were converted into barcoded cDNA libraries (NEBNext^®^ Multiplex Small RNA Library Prep Set, New England Biolabs, Ipswich, MA) using 400–500 ng of RNA as the starting material. Individual libraries prepared with unique indexes, were pooled and subjected to the Illumina sequencing pipeline, passing through clonal cluster generation on a single-read flow cell and 75 cycles sequencing-by-synthesis on the NextSeq 550 (Illumina Inc., San Diego, CA).

### Sequencing Data Analysis of Small RNA

The analysis process was automatized using the in-house developed customizable method as previously described (21). Briefly, raw reads were pre-processed by SeqTrimNext pipeline (http://www.scbi.uma.es/seqtrimnext) available at the Plataforma Andaluza de Bioinformática (University of Málaga, Spain) using the specific NGS technology configuration parameters. Subsequently, clean reads were aligned against human miRNA database (miRBase, release version 21), and Human Genome Assembly (GRCh37). Both, the CAP-miRSeq (21) and Oasis (22) pipeline were applied to identify human miRNA and to calculate their expression. The DE-miRNAs were determined by Log_2_ FC of EVOO vs. SO and FDR < 0.05 based on Benjamini and Hochberg multiple testing correction (23). miRNA-Seq for these experiments are publicly available in the NCBI BioProject database (PRJNA804481).

### Experimental Validation of miRNA Expression From EVs by RT-qPCR

The expression by RT-qPCR of 6 miRNAs in the same 8 samples (*n* = 4 per group) (technical validation) or in 16 samples (the same 4 samples per group plus another 4 samples per group) (biological validation) were analyzed on the basis of their ratio ranking and their biological interest. It was using the TaqMan^®^ MicroRNA Reverse Transcription kit following the manufacturer's instructions (Thermo Fisher Scientific Inc., Waltham, MA). miRNA expression was assessed by real-time PCR using an Applied Biosystems 7500 Fast Real-Time polymerase chain reaction System (Applied Biosystems, Foster City, CA). Reactions were carried out in duplicate for all miRNAs using TaqMan™ MicroRNA Assay (Thermo Fisher Scientific Inc., Waltham, MA): hsa-miR-1-3p (477820_mir), hsa-miR-216a-5p (477976_mir), hsa-miR-31-3p (478012_mir), hsa-miR-20a-5p (478586_mir), hsa-miR-126-5p (477888_mir), and hsa-miR-204-5p (478491_mir). The threshold cycle value for each sample was normalized with the expression of hsa-miR-1-3p (Ref: 477820_miR). We chose this miRNA because it was the endogenous control tested with a more constant expression (with the lowest coefficient of variation) and with greater expression. SDS software 2.3 and RQ Manager 1.2 (Applied Biosystems, Foster City, CA) were used to analyse the results with the comparative Ct method (2^−Δ*Ct*^). The Log_2_ FC of EVOO vs. SO were determined.

### Incubation of SMCs With EVs Secreted by HUVECs

Commercial human coronary artery smooth muscle cells (Cell Applications, Inc., San Diego, CA) were used, which were cultured with a SMCs growth medium (Cell Applications, INC, San Diego, CA) and penicillin/streptomycin (1%) at 37°C until reaching 90% confluence. Subsequently, differentiation was made into SMCs, obtaining these by culturing the former with SMC differentiation medium (Cell Applications, INC, San Diego, CA) and penicillin/streptomycin (1%) for 10 days. Differentiated SMCs were incubated with the EVs secreted by HUVECs obtained after incubation with the TRL from each patient and type of oil (n = 8/group). To do this, in a volume of 250 μL of M199 medium supplemented with exosome-depleted FBS (20%), glutamine (2 mM) and penicillin/streptomycin (1%), a volume of 100 μL of EVs was added and incubated at 37°C for 24 h. After this time, the SMCs were obtained for mRNA sequencing.

### Immunofluorescence in SMCs

EVs (*n* = 2 per group) were stained with PKH26 (PKH26 Red Fluorescent Cell Linker Kit for General Cell Membrane Labeling, PKH26GL-1KT) (Sigma-Aldrich, St. Louis, MO). The labeling of EVs with PKH26 was performed according to the manufacturer's instructions. Briefly, 1 ml of EVs was incubated with 1 ml of Solution PKH26 (1 ml of Diluent C + 4 uL of PKH26 ethanol solution) at room temperature for 5 min. The incubation was stopped with 2 ml of 1% BSA and centrifuged on Amicon^®^ Ultra-4 100 KDa centrifugal filters at 2,000 rpm for 10 min at 4°C to avoid free dye in the suspension and to eliminate the excess of dye in the EVs membrane. EV pellet was washed in PBS. This step was repeated at least twice, until the supernatant was transparent after the centrifugation and filtration. The staining of the EVs was done prior to the treatment of SMCs.

SMCs were plated, incubated and differentiated as above shown. SMCs were incubated with 70 μL of EVs stained with PKH26 in a total volume of 350 μL of M199 medium supplemented with exosome-depleted FBS (20%), glutamine (2 mM) and penicillin/streptomycin (1%). After 24 h, cells were washed three times with PBS and fixed with 4% paraformaldehyde for 10 min. Then, cells were permeabilized with 0.1% triton in PBS, washed and blocked with 3% BSA-PBS for 1 h at room temperature. After, cells were incubated overnight with a mouse monoclonal anti-α-smooth muscle actin antibody (A5228, Sigma-Aldrich, St. Louis, MO) (1:200 dilution) at 4°C, washed and incubated with Alexa Fluor™ 488 goat anti-mouse IgG 1/800 (A11029, Thermo Fisher Scientific Inc., Waltham, MA) as a secondary antibody for 1 h at room temperature. Finally, cell nuclei were counterstained with UltraCruz™ Mounting Medium for fluorescence with DAPI (Santa Cruz Biotechnology, Santa Cruz, CA) and analyzed with an Olympus BX51 microscope equipped with an Olympus DP70 digital camera (Olympus Europa SE & Co. KG, Hamburg, Germany). Analyses were performed in the IBIMA joint support structure for research (ECAI) of Microscopy.

### mRNA Extraction From SMCs

The mRNA of SMCs was isolated using Qiagen RNeasy Micro Kit (Qiagen Science, Hilden, Germany) as described by the manufacturer and was reverse transcribed using High-Capacity cDNA Reverse Transcription Kit 200 reactions (Ref 4368814, Thermo Fisher Scientific Inc., Waltham, MA) and RNase Inhibitor Set (Ref N8080119, Thermo Fisher Scientific Inc., Waltham, MA).

### Library Preparation and mRNA Sequencing

Poly-A enriched strand-specific libraries were generated with the TruSeq Stranded mRNA (Illumina, Inc., San Diego, CA) sample preparation following the manufacturer's instructions, using 400–500 ng of RNA as the starting material. Library pools were sequenced on a Nextseq550 instrument (Illumina Inc., San Diego, CA) with a pair-end sequencing (2 × 76 cycles) on a High-Output flow-cell, following the manufacturer's instructions.

### Sequencing Data Analysis of mRNA

The analysis process was automatized using the in-house developed customizable method. Raw reads were pre-processed by SeqTrimNext pipeline (http://www.scbi.uma.es/seqtrimnext) (24) available at the Plataforma Andaluza de Bioinformática (University of Málaga, Spain) using the specific NGS technology configuration parameters. This pre-processing removes low quality, ambiguous and low complexity stretches, adaptors, organelle DNA, polyA/polyT tails, and contaminated sequences while keeping the longest (at least >20 bp) informative part of the read. Subsequently, the useful reads were aligned against Human Genome Assembly GRCh38 (hg38) using STAR v2.5 (25). The alignment file was used by Cufflinks (v.2.2.1) (24) followed by Cuffquant and then Cuffdiff, for assessing expression levels of genes as previously described (26). CummeRbund v3.6 was then pipelined to analyze, explore, manipulate and visualize the results. DE-mRNA were determined by Log_2_ FC of EVOO vs. SO using as filters an adjusted *P* < 0.05. mRNA-Seq for these experiments are publicly available in the NCBI BioProject database (PRJNA804481).

### GO and KEGG Analysis

GSEA was used (MSigDB database v7.2 updated September 2020; http://software.broadinstitute.org/gsea/index.jsp) (27, 28) to obtain the GO and the KEGG of the DE-mRNA of the SMCs.

### Experimental Validation of mRNA Expression From SMCs Using RT-qPCR

Four gene expression levels were analyzed in duplicate in the same samples (*n* = 4 per group) (technical validation) or in 8 samples (the same 4 samples per group plus another 4 samples per group) (biological validation) by quantitative real-time reverse transcriptase-PCR using ABI 7500 Fast Real-Time PCR System (Applied Biosystems, Foster City, CA, USA). They were analyzed on the basis of their ratio ranking (a higher Fold Change) and their biological interest (those that could be related to related to endothelial dysfunction and atherosclerosis). RT qPCR reactions were carried out for the following genes using specific TaqMan™ Gene Expression Assays (Thermo Fisher Scientific Inc., Waltham, MA): MAPK10 (Hs00959260_m1), SLC8A1 (Hs01062258_m1), CCND1 (Hs00765553_m1), and ABCA8 (Hs00200350_m1). The threshold cycle (Ct) value for each sample was normalized with the expression of B2M (Hs99999907_m1). The mRNA level expression was determined by Log_2_ FC of EVOO vs. SO.

### Interaction Between mRNA From SMCs and miRNA From EVs by IPA and GSEA

The possible interaction between the DE-mRNAs from SMCs and the DE-miRNAs from EVs were analyzed using the web-based bioinformatic tool IPA (Ingenuity Systems, http://www.ingenuity.com). All up and downregulated DE-miRNAs from EVs and DE-mRNAs from SMCs, as well as FC, were imported into IPA. Interactions were predicted by using the different databases integrated in IPA software (miRecords, Tarbase and TargetScan Human). Gene Set Enrichment Analysis (GSEA) was used (GSEA; GSEA/MSigDB web site v6.4 version; MSigDB database v7.0 updated August 2019; http://software.broadinstitute.org/gsea/index.jsp) (27) to obtain the Gene Ontology (GO) of the predicted interactions.

## Results

### Characterization of EVs Secreted by HUVECs

**Figure 1:**
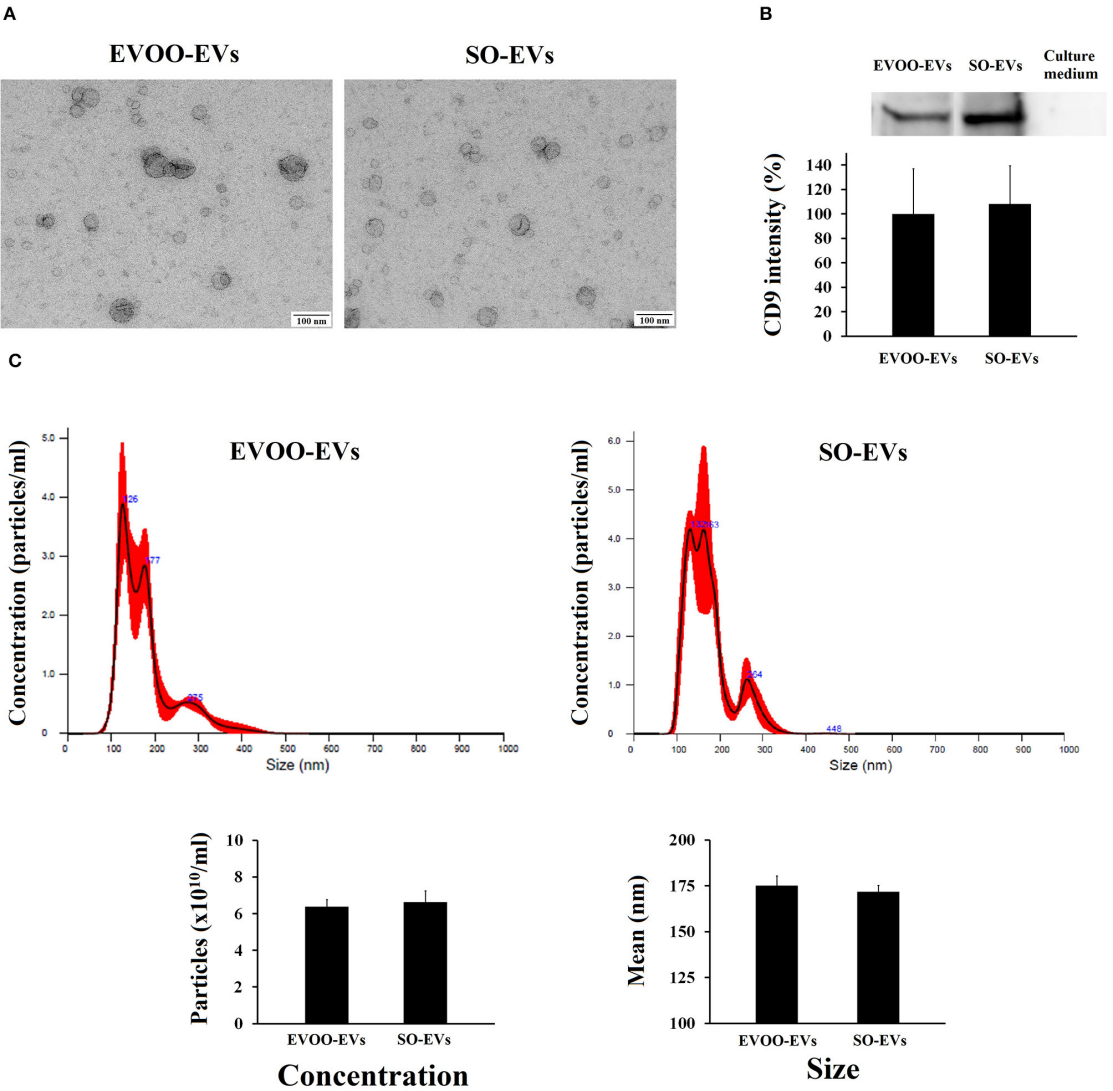
**(A)** Characterization by electronic microscopy of the EVs secreted by HUVECs incubated with EVOO-derived TRLs (EVOO-EVs) and with SO-derived TRLs (SO-EVs). **(B)** Graph shows CD9 in the EVOO-EVs, in the SO-EVs and in the culture medium supplemented with exosome-depleted FBS used in the incubations, with representative western blot above for CD69 (*n* = 2). **(C)** Analysis of the concentration and size distribution of EVs by nanoparticle tracking analysis (*n* = 2).

First, we characterized the EVs secreted by HUVECs incubated with the TRLs obtained 3 h after an HF meal with EVOO (EVOO-EVs) and SO (SO-EVs). Figure 1A shows the typical morphology of exosomes obtained by electronic microscopy. Figure 1B shows the western blot of CD9 protein, characteristic of EVs, in EVOO-EVs, SO-EVs and in a sample of the culture medium used for the incubations. No significant differences were found between EVOO-EVs and SO-EVs (*p* = 0.667). As can be seen, this culture medium was supplemented with exosome-depleted FBS since no CD9 signal was found. We also did not find significant differences in the size (*p* = 0.186) and concentration (*p* = 0.958) of EVOO-EVs and SO-EVs by nanoparticle tracking analysis (Figure 1C).

### DE-miRNAs Between EVOO and SO in EVs

**Figure 2:**
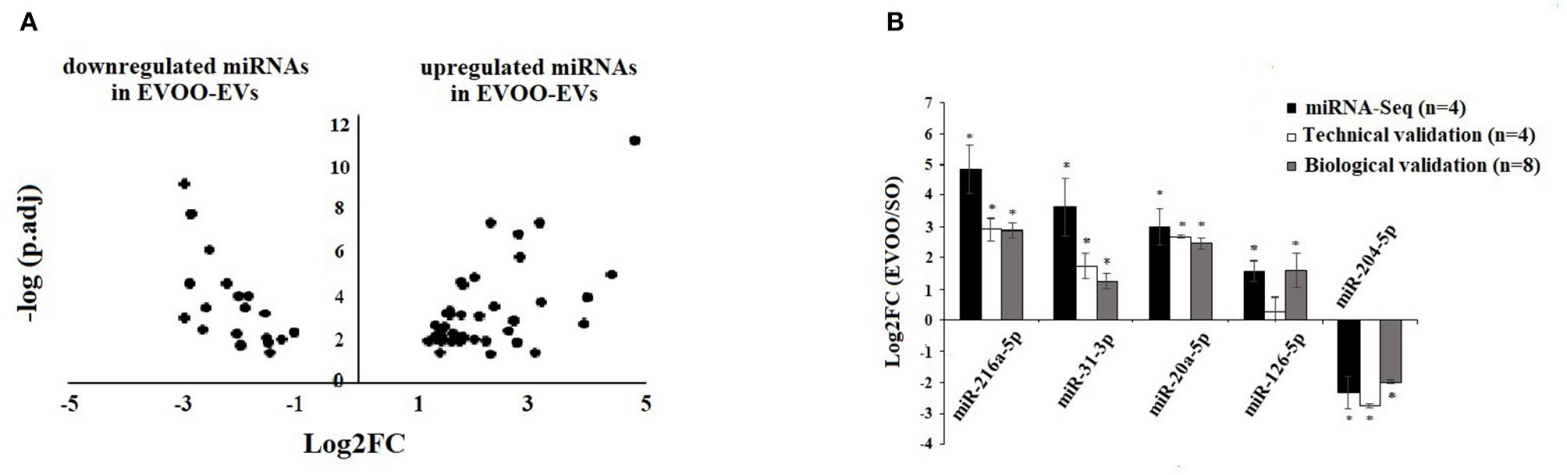
**(A)** Volcano Plot of the DE-miRNAs in EVs secreted by HUVECs incubated with EVOO-derived TRLs (EVOO-EVs) and SO-derived (SO-EVs) TRLs. Positive Log2FC: upregulated miRNAs in EVOO-EVs. Negative Log2FC: downregulated miRNAs in EVOO-EVs. **(B)** Technical and biological validation of 4 up and 1 downregulated DE-miRNA obtained in EVOO-EVs vs. SO-EVs. Data from miRNA-Seq (in black) (*n* = 4 per group), from technical validation [in the same samples (*n* = 4 per group)] (in white), and from biological validation (in the same 4 samples per group plus another 4 samples per group) (in gray). Data are represented as the mean ± SEM. Log2FC: Fold Change in the expression of each gene. *P < 0.05: significant Log2FC in the up/downregulated miRNAs.

We evaluated with both CAP-miRSeq and Oasis miRNA analysis, the profile of DE-miRNAs in EVs secreted by HUVECs incubated with the TRLs obtained 3 h after an HF meal with EVOO and SO. We found 125 DE-miRNAs using Oasis and 110 DE-miRNAs using CAP-miRSeq, with a total of 60 DE-miRNAs common between the two analyses. From those 60 DE-miRNAs, 41 were upregulated and 19 downregulated in EVOO-EVs (Supplementary Table 2, Figure 2A).

### Validation of DE-miRNA Obtained From Sequencing From EVs Secreted by HUVECs

We found a similar behavior in the data obtained from miRNA-Seq (*n* = 4 per group), from RT-qPCR in the same samples (*n* = 4 per group) (technical validation) and from RT-qPCR in 8 samples (the same 4 samples per group plus another 4 samples per group) (biological validation) (Figure 2B). We found a significant (*p* < 0.05) upregulation of miR-216a-5p, miR-31-3p, miR-20a5p, and miR-126-5p and a downregulation of miR-204-5p in EVOO-EVs.

### DE-mRNAs in SMCs Incubated With EVOO-EVs and SO-EVs

**Figure 3:**
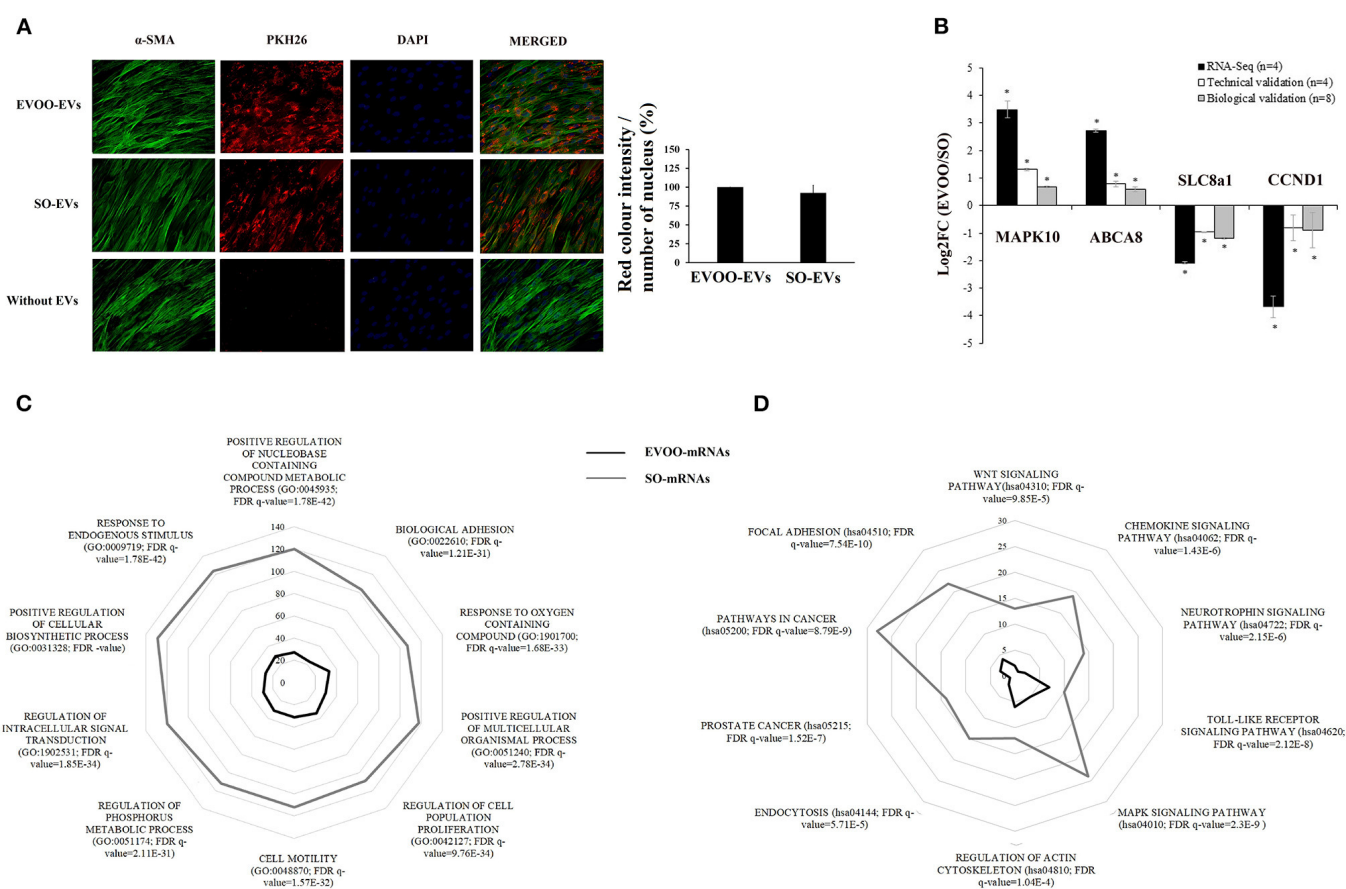
**(A)** Microscopy analysis of EVs internalization. SMCs were incubated for 24h with EVOO-EVs and SO-EVs dyed with PKH26. Negative control without EVs is also shown. Analysis of the intensity of red color (EVs)/number of nucleus is also shown (*n* = 2). **(B)** Technical and biological validation of 2 up and 2 downregulated DE-mRNA obtained from SMCs incubated with EVOO-EVs and SO-EVs. Data from mRNA-Seq (in black) (*n* = 4 per group), from technical validation [in the same samples (*n* = 4 per group)] (in white), and from biological validation (in the same 4 samples per group plus another 4 samples per group) (in gray). Data are represented as the mean ± SEM. Log2FC: Fold Change in the expression of each gene. *P < 0.05: significant Log2FC in the up/downregulated mRNAs. **(C)** Number of DE-mRNAs of EVOO-mRNAs (black) and SO-mRNAs (gray) involved in the 10 most differentially expressed GO:BP. **(D)** Number of DE-mRNAs of EVOO-mRNAs (black) and SO-mRNAs (gray) involved in the 10 most significant KEGG pathways.

First, we checked that both EVOO-EVs and SO-EVs were internalized by SMCs (Figure 3A). We did not find significant differences between EVOO-EVs and SO-EVs internalization [intensity of red color (EVs)/number of nucleus] (*p* = 0.543, Figure 3A). After, we evaluated the profile of DE-mRNAs in the SMCs (DE-mRNAs-SMC) incubated with the EVOO-EVs and SO-EVs. We found 991 DE-mRNA-SMC, of which 277 were upregulated (EVOO-mRNAs) and 714 were downregulated in SMCs incubated with EVOO-EVs or, what is the same, were upregulated in SMCs incubated with SO-EVs (SO-mRNAs). These genes are described in Supplementary Table 3.

### Validation of the DE-mRNA of SMCs Obtained From Sequencing

We found a similar behavior in the data obtained from miRNA-Seq (*n* = 4 per group), from RT-qPCR in the same samples (*n* = 4 per group) (technical validation) and from RT-qPCR in 8 samples (the same 4 samples per group plus another 4 samples per group) (biological validation) (Figure 3B). Two of them were upregulated in EVOO-mRNAs (MAPK10 and ABCA8), and another 2 were downregulated (SLC8a1 and CCND1).

### GO Analysis of the DE-mRNA From SMCs

We found a lot of GO terms with the DE-mRNA from SMCs. We analyzed the 10 most differentially expressed GO terms related to the BP (GO:BP) found in the DE-mRNAs of SMCs. These 10 GO:BP were: response to endogenous stimulus (GO:0009719), positive regulation of cellular biosynthetic process (GO:0031328), regulation of intracellular signal transduction (GO:1902531), positive regulation of multicellular organismal process (GO:0051240), regulation of phosphorus metabolic process (GO:0051174), cell motility (GO:0048870), regulation of cell population proliferation (GO:0042127), response to oxygen-containing compound (GO:1901700), biological adhesion (GO:0022610) and positive regulation of nucleobase-containing compound metabolic process (GO:0045935). Figure 3C shows that the number of genes of each GO:BP was higher in the SO-mRNA group. Supplementary Table 4 shows the EVOO-mRNAs and SO-mRNAs involved in each GO:BP.

### KEGG Analysis of the DE-mRNA From SMCs

We analyzed the 10 most differentially expressed KEGG pathways in which the DE-mRNAs were involved. These KEGG pathways were: focal adhesión (hsa04510), pathways in cancer (hsa05200), prostate cancer (hsa05215), endocytosis (hsa04144), regulation of actin cytoskeleton (hsa04810), MAPK signaling (hsa04010), toll-like receptor signaling (hsa04620), neurotrophin signaling (hsa04722), chemokine signaling (hsa04062) and Wnt signaling (hsa04310). Figure 3D shows that the number of genes of each KEGG pathway was higher in the SO-mRNA group. Supplementary Table 5 shows the EVOO-mRNAs and SO-mRNAs involved in each KEGG pathway selected.

### Interaction Between DE-miRNA From EVs and DE-mRNA From SMCs

**Figure 4:**
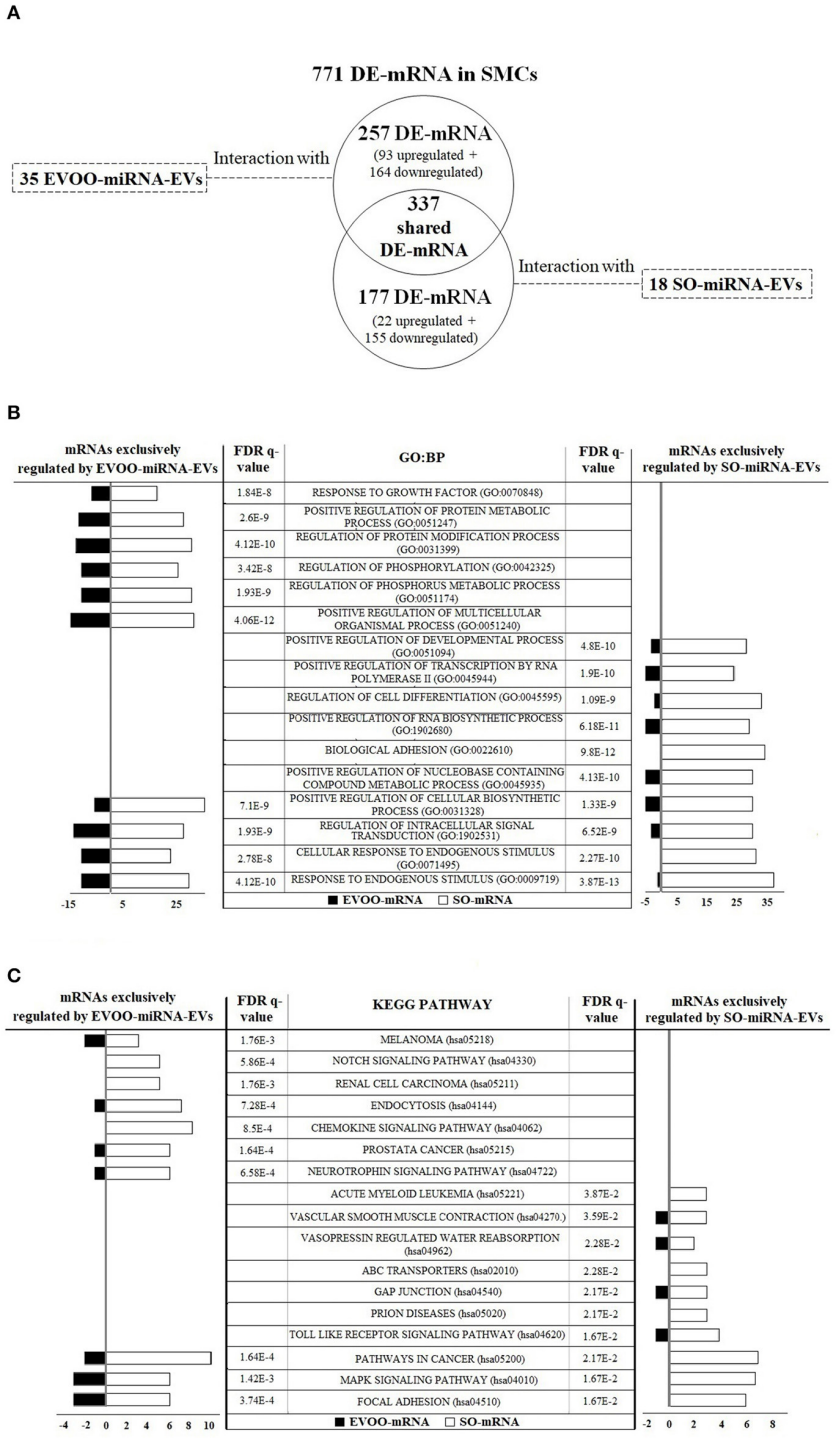
**(A)** The interaction between the 771 DE-mRNAs found in SMCs and the 53 DE-miRNAs found in EVs was analyzed. Thirty-five upregulated DE-miRNA obtained from EVs secreted by HUVECs incubated with EVOO-derived TRLs (EVOO-miRNA-EVs) could exclusively regulate 257 DE-mRNAs in SMCs (93 up and 164 downregulated EVOO-mRNAs). Meanwhile, 18 upregulated DE-miRNA obtained from EVs secreted by HUVECs incubated with SO-derived TRLs (SO-miRNA-EVs) could exclusively regulate 177 DE-mRNAs in SMCs (22 up and 155 downregulated SO-mRNAs). But 337 DE-mRNAs could be regulated by both EVOO-miRNA-EVs and SO-miRNA-EVs. **(B)** GO:BP terms found in the 257 DE-mRNA from SMCs that could be exclusively regulated by EVOO-miRNA-EVs and in the 177 DE-mRNA from SMCs that could be exclusively regulated by SO-miRNA-EVs. **(C)** KEGG pathways found in the 257 DE-mRNA from SMCs that could be exclusively regulated by EVOO-miRNA-EVs and in the 177 DE-mRNA from SMCs that could be exclusively regulated by SO-miRNA-EVs.

Using IPA software, the interaction between the 991 DE-mRNAs from SMCs and the 60 DE-miRNAs from EVs was analyzed. IPA software found an interaction between 771 DE-mRNAs and 53 DE-miRNAs (35 EVOO-miRNA-EVs and 18 SO-miRNA-EVs) (Figure 4A). Three hundred and thirty-seven DE-mRNAs interacted with both EVOO-miRNA-EVs and SO-miRNA-EVs. Two hundred and fifty-seven DE-mRNAs exclusively interacted with the 35 EVOO-miRNA-EVs (93 up and 164 downregulated in EVOO-mRNA). And 177 DE-mRNA exclusively interacted with the 18 SO-miRNA-EVs (22 up and 155 downregulated in EVOO-mRNA). Some of these interaction were experimentally observed in other studies (Supplementary Table 6).

We analyzed the 10 most significant GO:BP terms found in the 257 DE-mRNA from SMCs that could be exclusively regulated by EVOO-miRNA-EVs and in the 177 DE-mRNA from SMCs that could be exclusively regulated by SO-miRNA-EVs (Figure 4B). We found 6 exclusive GO:BP in the interaction between DE-mRNA and EVOO-miRNA-EVs [response to growth factor (GO:0070848), positive regulation of protein metabolic process (GO:0051247), regulation of protein modification process (GO:0031399), regulation of phosphorylation (GO:0042325), regulation of phosphorus metabolic process (GO:0051174), and positive regulation of multicellular organismal process (GO:0051240)], 6 exclusive GO:BP in the interaction between DE-mRNA and SO-miRNA-EVs [positive regulation of developmental process (GO:0051094), positive regulation of transcription by RNA polymerase II (GO:0045944), regulation of cell differentiation (GO:0045595), positive regulation of RNA biosynthetic process (GO:1902680), biological adhesion (GO:0022610) and positive regulation of nucleobase containing compound metabolic process (GO:0045935)], and 4 shared GO:BP [positive regulation of cellular biosynthetic process (GO:0031328), regulation of intracellular signal transduction (GO:1902531), cellular response to endogenous stimulus (GO:0071495) and response to endogenous stimulus (GO:0009719)].

Likewise, we analyzed the 10 most significant KEGG pathways found in the 257 DE-mRNA from SMCs that could be exclusively regulated by EVOO-miRNA-EVs and in the 177 DE-mRNA from SMCs that could be exclusively regulated by SO-miRNA-EVs (Figure 4C). In each group, we found 7 exclusive KEGG pathways and 3 shared KEGG pathways (Figure 4C). We found 7 exclusive KEGG pathways in the interaction between DE-mRNA and EVOO-miRNA-EVs [melanoma (hsa05218), notch signaling (hsa04330), renal cell carcinoma (hsa05211), endocytosis (hsa04144), chemokine signaling (hsa04062), prostate cancer (hsa05215) and neurotrophin signaling (hsa04722)]. We found 7 exclusive KEGG pathways in the interaction between DE-mRNA and SO-miRNA-EVs [acute myeloid leukemia (hsa05221), vascular smooth muscle contraction (hsa04270), vasopressin regulated water reabsorption (hsa04962), ABC transporters (hsa02010), gap junction (hsa04540), prion diseases (hsa05020) and toll like receptor signaling (hsa04620)]. We also found 3 shared KEGG pathways [cancer (hsa05200), MAPK signaling (hsa04010) and focal adhesion (hsa04510)].

## Discussion

In this study, we have shown for the first time that the miRNA composition of EVs secreted by HUVECs is different in response to the TRLs derived from two types of oils normally used in the diet (EVOO and SO). Moreover, the EVs secreted from HUVECs incubated with TRL derived from SO intake produced significant changes in the SMC phenotype with regard to those derived from EVOO intake, with a profile more associated with the development of atherosclerosis.

Previous studies on endothelial cells have shown that this type of cells can modify the release of EVs and their content in response to different cardiovascular risk factors, such as TNFα and angiotensin II (14, 16, 17). We have not analyzed in depth in our study the amount of EVs/cells that HUVECs secreted. This gives us an interesting point of view to expand our future research in this field. However, our study we went further. We studied how the miRNA composition of HUVEC-derived EVs was modified in response to diets with a different fat composition. These EVs with different composition could be producing different effects on other cell types. In this context, the interrelationship between endothelial and SMCs can be relevant in the development of atherosclerosis (11). Previous studies have shown that endothelial cell-derived EVs could directly regulate the SMC phenotype (13, 14). Also, the EVs secreted by HUVECs incubated with high concentrations of glucose induced mitochondrial dysfunction in vascular SMCs (29). Moreover, other studies in ApoE^(−/−)^ mice have shown the atheroprotective capacity of endothelial cell-derived EVs on vascular SMCs through their miRNA content (13, 30).

In our study, we found that the EVs secreted by HUVECs in response to TRLs derived from EVOO and SO intake produced a different profile of mRNA expression in SMCs. SO-EVs were more associated with a higher induction of pathways related to cell migration and motility than EVOO-EVs (focal adhesion, cytoskeleton actin regulation, MAPK signaling and neurotrophin pathways, and cell motility and regulation of cell population proliferation). In this context, an abnormal proliferation and apoptosis is the pathological basis of atherosclerosis-related diseases (31). MAPKs are kinases primarily involved in the regulation of cell proliferation, differentiation, migration, and activate apoptotic cell death (32). These kinases, such as MAPK10 are upregulated with EVOO-EVs, control the cellular response to a damaging extracellular stimulus. However, when the damage produced cannot be repaired, the cell must be reprogrammed to die (apoptosis). In this type of cell death, p53 plays an important role. P53 (also known as TP53) activation would subsequently occur to promote cell apoptosis. Our results with SO-EVs also show a significant increase in the expression of this gene. This increase in p53 with SO-EVs could be the response to a greater alteration or cellular damage produced by these SO-EVs compared to that produced by EVOO-EVs. This p53 is a tumor suppressor protein that responds to increased cellular stress to regulate the expression of target genes, thus inducing the progress of cardiovascular diseases *via* anti-angiogenesis, pro-apoptosis, pro-autophagy, pro-necrosis, metabolism regulation, and cell cycle arrest regulation (33).

Other pathways more induced by SO-EVs than by EVOO-EVs are related to endocytosis and the regulation of signal transduction (Wnt and MAPK signaling pathway), and especially related to inflammation [Toll-like receptor (TLR) and chemokine signaling pathway]. The Wnt signaling pathway is required for basic developmental processes, such as the proliferation of progenitor cells and the control of asymmetric cell division. There were are many genes overexpressed with SO-EVs in the canonical Wnt/ß-catenin and Wnt/Ca^2+^ pathways, such as PKA (or PRKACA), NKD, p53, CBP (or CREBBP) and PKC (or PRKACA). In these pathways, the mRNA expression of co-receptors LRP5, nuclear TCFs, CaMKII, which are required to regulate Wnt target gene transcription (34, 35), were also increased with SO-EVs. Moreover, the induction of endothelial dysfunction by the activation of Wnt/ß-catenin can be produced by TGFB (36), other gene overexpressed with SO-EVs. These proteins are involved in the pathogenesis of vascular damage, after that, vascular SMCs modify their phenotype from the baseline contractile state to a more proliferative state. This phenotypic switch allows its migration to subintima, where they proliferate and deposit an extracellular matrix and contribute to atherosclerosis formation (37). In addition, we have found that cyclin D1, a protein involved in the increased pro-proliferative effect of ß-catenin on VSMCs (38), is inhibited with EVOO-EVs. However, we do not have data on SMCs proliferation to confirm the possible change in SMCs phenotype from contractile to proliferative state, a key feature for the development of atherosclerosis. This is a limitation of the study.

In the regulation of inflammation, TLRs play an important role by inducing the production of proinflammatory cytokines and co-stimulatory molecules (39). They recognize stimuli from both external pathogens and endogenous cell damage (40). Our results showed that this pathway was more stimulated with SO-EVs. These type of EVs produced an upregulation of several genes such as Nf-kβ, MAPK and IL-1B, which are involved in the MyD88-dependent TLR pathway. Nf-kβ is involved in proliferation, inflammation and anti-apoptosis processes, and increases JNK. IL-1B is one of the main proinflammatory cytokines capable of inducing changes in vascular SMC morphology associated with the progression of atherosclerosis (41). Also, we found that the upregulation of IL-1B and Nf-kβ is accompained by an upregulation of IRAK1. It is known that in atherosclerosis there is an activation of the inflammatory cascade mediated by the TLR pathway through IRAK1/Nf-kβ (42).

In addition to studying the direct effects of the EVs secreted by HUVECs on SMCs, and according to the IPA program, we found that some of the DE-mRNAs from SMCs could be regulated by miRNAs from EVs. There are some mRNAs that could be exclusively regulated by miRNAs upregulated in EVOO-EVs and others mRNAs exclusively regulated by miRNAs downregulated in EVOO-EVs. The mRNAs involved in these interactions participate in GO and pathways similar to those found for the DE-mRNAs obtained in SMCs. Those mRNAs that interact with miRNAs downregulated by EVOO-EVs, or what is the same upregulated by SO-EVs, appear to be involved in processes related to biological adhesion, cell differentiation and development, as well as RNA transcription, and in pathways related to atherosclerosis, such as inflammation, vascular smooth muscle contraction, ABC transporters, and GAP junctions. ABC transporters form one of the most important families of substrate transporters, such as lipids, and are closely related to atherosclerosis (43, 44). Also, the intercellular communication due to GAP junctions is essential for many physiological events, such as embryonic development, metabolite transport, apoptosis, and play an important role in the development of atherosclerosis (45). These junctions seem to play a relevant role in the phenotypic transformation of vascular SMCs from the differentiated contractile state to the synthetic activated state, which is related to an increase in the migration and proliferation of vascular SMCs, and associated with the development of atherosclerosis. On the other hand, there is also a group of mRNAs that appears to be exclusively regulated by miRNAs upregulated by EVOO-EVs related to chemokine signaling, endocytosis, and Notch signaling pathway, which is another intercellular communication mechanism. However, there are also a large number of mRNAs in SMCs that could be regulated by both, miRNAs upregulated and downregulated in EVOO-EVs. Their effects on the mRNA expression would be the result of the sum of all the possible effects of the miRNAs present in the EVs.

This study shows how diet could be modulating intercellular communication through a different miRNA composition of EVs secreted by endothelial cells. However, we did not have enough sample to carry out another type of study or increase the number of samples for each of the analyzes that we have carried out. This is another limitation of our study. But we have to keep in mind that the possible effects of EVs could also be mediated by other molecules contained in EVs, such as proteins and lipids (46). Although it would be interesting to show data on the proteomic or lipid profile of the EVs used, these analysis were not performed in our study. EVs from endothelial cells subjected to a proinflammatory stimulus have a lower amount of TET2, which, after transfer to vascular SMCs, promotes a change in their phenotype leading to the formation of atheroma plaque and the development of atherosclerosis (46). Moreover, it would be interesting to directly analyze in a later study the metabolic changes produced by these endothelial cells-derived EVs on SMCs to confirm the results obtained with the transcriptomic profile.

In conclusion, we found that endothelial cells can affect SMCs transcriptomic profile through the EVs they secrete. These EVs have a different miRNA composition depending on the stimulus to which they are subjected, in our case two oils of different composition, EVOO and SO. EVOO is a main component of the Mediterranean diet, which has been associated in numerous epidemiological studies with a lower incidence of cardiovascular disease. This different miRNA composition of HUVEC-derived EVs could be involved in the beneficial effect of EVOO on atherosclerosis through a better regulation of SMCsmetabolism.

## Data Availability Statement

The datasets presented in this study can be found in online repositories. The names of the repository/repositories and accession number(s) can be found below: NCBI BioProject database (Accession Number: PRJNA804481).

## Author Contributions

CS-F, EG-F, and LG-S: conceptualization, formal analysis, and writing—original draft preparation. CS-F, CR-D, AH-P, CG-R, WO-O, and FM-R: methodology. RB and JG-M: software. CS-F, AH-P, and CG-R: validation. MT, EG-F, and LG-S: investigation. LG-S, EG-F, and FT: resources. CS-F, FT, EG-F, and LG-S: writing—review and editing. EG-F and LG-S: supervision. LG-S: funding acquisition. All authors have read and agreed to the published version of the manuscript.

## Funding

This work was supported in part by Grants from the Instituto de Salud Carlos III (PI14/01306) (Spain). This study has been co-funded by FEDER funds (A way to make Europe). CIBER Fisiopatología de la Obesidad y Nutrición (CIBEROBN) and CIBER de Enfermedades Hepáticas y Digestivas (CIBEREHD) are ISCIII projects. LG-S was supported by the Miguel Servet program from the ISCIII (Spain) (Miguel Servet II Program, CPII18/00030) and Nicolas Monardes program from the Consejería de Salud de Andalucía (Spain) (C-0028-2018). CS-F was supported by a Grant from the ISCIII (Spain) (PFIS Program, FI16/00241). CR-D was supported by a Grant from ISCIII (Spain) (CD18/00188). CG-R was supported by the Miguel Servet program from the ISCIII (Spain) (Miguel Servet Program, CP20/00066). FM-R was supported by a Grant from the ISCIII (Spain) (PFIS program, FI19/00189). AH-P was supported by a Grant from the Consejeria de Salud de la Junta de Andalucía (Spain) (PE-0098-2019). EG-F was supported by the Nicolas Monardes Program from the Consejería de Salud de Andalucía (Spain) (C-0031-2016).

## Conflict of Interest

The authors declare that the research was conducted in the absence of any commercial or financial relationships that could be construed as a potential conflict of interest.

## Publisher's Note

All claims expressed in this article are solely those of the authors and do not necessarily represent those of their affiliated organizations, or those of the publisher, the editors and the reviewers. Any product that may be evaluated in this article, or claim that may be made by its manufacturer, is not guaranteed or endorsed by the publisher.
