# Supplementary material for: EVOO Promotes a Less Atherogenic Profile Than Sunflower Oil in Smooth Muscle Cells Through the Extracellular Vesicles Secreted by Endothelial Cells
Source: Front Nutr. 2022 Apr 12;9:867745. doi: 10.3389/fnut.2022.867745 (PMC9039400; doi:10.3389/fnut.2022.867745)
Supplement: Supplementary file 1 [file Table_1.DOCX]

**Supplementary Table 1.** Anthropometric and biochemical variables in patients included in the study.

|  | **With EVOO (n=8)** | **With SO (n=8)** | ***P*** |
| --- | --- | --- | --- |
| **Sex (Male/Female)** | 3/5 | 3/5 |  |
| **Age (years)** | 30.1±7.8 | 31.0±6.6 | 0.564 |
| **Weight (kg)** | 61.3±6.7 | 65.5±7.1 | 0.253 |
| **BMI (kg/m^2^)** | 21.8±1.5 | 22.7±2.0 | 0.317 |
| **Waist (cm)** | 79.9±5.9 | 81.8±6.3 | 0.510 |
| **Hip (cm)** | 96.1±4.5 | 99.9±6.9 | 0.288 |
| **Glucose(mg/dl)** | 74.8±9.0 | 77.6±11.3 | 0.583 |
| **Cholesterol (mg/dl)** | 191.8±41.6 | 186.5±41.4 | 0.804 |
| **Triglycerides (mg/dl)** | 84.3±26.1 | 78.0±29.9 | 0.663 |
| **HDL (mg/dl)** | 59.6±11.5 | 62.4±13.9 | 0.672 |
| **LDL (mg/dl)** | 114.9±40.7 | 108.2±39.5 | 0.743 |

BMI: Body mass index.

**Supplementary Table 2**. DE-miRNAs common between the two analysis (Oasis and Cap-miRSeq).

| **DE-miRNAs upregulated in**  **EVs secreted by HUVECs**  **incubated with EVOO-TRLs** | | | | **DE-miRNAs downregulated in EVs secreted by HUVECs incubated with EVOO-TRLs** | |
| --- | --- | --- | --- | --- | --- |
| hsa-let-7g-5p | hsa-miR-106b-5p | hsa-miR-125b-2-3p | hsa-miR-126-5p | hsa-miR-127-3p | hsa-miR-128-3p |
| hsa-miR-1271-5p | hsa-miR-1307-5p | hsa-miR-132-3p | hsa-miR-140-3p | hsa-miR-130b-5p | hsa-miR-143-3p |
| hsa-miR-148b-3p | hsa-miR-16-5p | hsa-miR-181b-5p | hsa-miR-181d-5p | hsa-miR-183-5p | hsa-miR-1908-5p |
| hsa-miR-195-5p | hsa-miR-19a-3p | hsa-miR-19b-3p | hsa-miR-20a-5p | hsa-miR-204-3p | hsa-miR-204-5p |
| hsa-miR-216a-3p | hsa-miR-216a-5p | hsa-miR-217 | hsa-miR-23a-3p | hsa-miR-222-3p | hsa-miR-323a-5p |
| hsa-miR-27b-3p | hsa-miR-301b-3p | hsa-miR-30b-5p | hsa-miR-30c-5p | hsa-miR-409-3p | hsa-miR-411-3p |
| hsa-miR-31-3p | hsa-miR-31-5p | hsa-miR-3200-3p | hsa-miR-324-3p | hsa-miR-424-3p | hsa-miR-4488 |
| hsa-miR-345-5p | hsa-miR-361-5p | hsa-miR-425-3p | hsa-miR-425-5p | hsa-miR-485-3p | hsa-miR-485-5p |
| hsa-miR-452-5p | hsa-miR-574-3p | hsa-miR-582-3p | hsa-miR-641 | hsa-miR-543 | hsa-miR-615-3p |
| hsa-miR-652-3p | hsa-miR-671-5p | hsa-miR-874-3p | hsa-miR-887-3p | hsa-miR-671-3p |  |
| hsa-miR-93-5p |  |  |  |  |  |

**Supplementary Table 3.** DE-mRNAs in SMCs incubated with EVOO-EVs and SO-EVs.

| **DE-mRNAs upregulated in SMCs incubated with EVOO-EVs** | | | | | | |
| --- | --- | --- | --- | --- | --- | --- |
| A2M | ABCA8 | AC018647.3 | AC104654.2 | ACSS1 | ACTR6 | ADAMTS3 |
| AKAP6 | AKR1C3 | AMZ2P1 | ANG,ARNSE4 | ANXA3 | ARHGAP20 | ARL4A |
| ARRDC3 | ASNS | ATP10D | ATP8B4 | BBS10 | BBS12 | BBS9 |
| BCHE | BDH2 | C1orf63 | C2orf27A | C2orf76 | CACNB4 | CALCRL |
| CAND1 | CARD16 | CCDC53 | CCDC7 | CCNG1 | CD14 | CDH8 |
| CEP112 | CEP70 | CLK1 | CMAHP | CMYA5 | CNKSR2 | COL14A1 |
| COX7C | CRISPLD1 | CSRP2 | CSTA | CTNNAL1 | CTSK | CWF19L2 |
| CYBRD1 | DDIT4L | DDX60 | DECR1 | DHRS3 | DNM3OS | DOCK11 |
| DPYD | DTNA | DYNC2H1 | EDNRB | EEA1 | EFHC1 | EIF3E |
| EIF4A2 | EMB | EMC2 | ESD | F2R | FAM102B | FAM129A |
| FAM13C | FAM171B | FAM46C | FAM49A | FAP | FBXO25 | FBXO3 |
| FBXO4 | FGF7 | FIBIN | FLJ35282 | FMN1 | FMO1 | FMO2 |
| FMO3 | FMO4 | FNBP1L | FRK | FRRS1 | FXR1 | GABRE |
| GAS5 | GBP2 | GCNT4 | GEM | GLS | GLT8D2 | GPC6 |
| GPNMB | GPR126 | GSG1 | GSTA4 | GTF2A1L, STON1,  STON1-GTF2A1L | GTF2H5 | GUCY1A2 |
| GYG1 | HAT1 | HCFC2 | HLTF | HNMT | HNRNPLL | HSD17B6 |
| ID4 | IER5L | IFIT1 | IFIT2 | IFT52 | IGJ | IGSF10 |
| IKZF2 | INTS8 | INTU | IRAK4 | ITGA4 | KCNE4 | KCTD16 |
| KCTD20 | KIAA1009 | KIAA1107 | KIAA1109 | KIAA1324L | KIAA1731 | KLHL4 |
| KRCC1 | LAMA2 | LCA5 | LDB2 | LGALS3 | LIMCH1 | LINC01018 |
| LMBRD1 | LOC100131564 | LOC100506060 | LOC100506870 | LOC100507165 | LOC100507460 | LOC101929690 |
| LOC102724783 | LOC653513 | LRRC17 | LRRK2 | LSMEM1 | LUM | LXN |
| LY96 | MAN1C1 | MAPK10 | MBOAT2 | MCU | MEIS1 | MFSD1 |
| MGARP | MGP | MIAT | MLF1 | MORN2 | MSR1 | MTUS1 |
| MYH10 | MYH15 | N4BP2L2 | NAALADL2 | NDUFS4 | NEDD1 | NFYB |
| NOX4 | NUP37 | OGT | OMA1 | OSBPL8 | OSR2 | PABPC4L |
| PABPC5 | PCDH18 | PCMTD1 | PDE1A | PDE5A | PDGFD | PDGFRL |
| PDLIM3 | PIGK | PKIB | PLA2G16 | PLCL1 | PLK2 | PLS3 |
| PLSCR1 | PLSCR4 | PLXDC2 | PPP1R3C | PRCP | PROS1 | PRSS35 |
| PRTFDC1 | PRUNE2 | PTK2B | PTPLAD2 | RARRES1 | RBM43 | RN7SL1 |
| RN7SL2 | RNF112 | ROR1 | RP11-417E7.2 | RP6-206I17.2 | RPL39 | RPL7 |
| RPL9 | RSPO3 | RWDD3, TMEM56,  TMEM56-RWDD3 | SCN9A | SCP2 | SCRG1 | SEPP1 |
| SERAC1 | SERPINB7 | SESN3 | SKP1P2 | SLC16A4 | SLC38A2 | SLC40A1 |
| SLC44A5 | SLC9A9 | SMARCA1 | SMC6 | SMPDL3A | SMYD3 | SNHG5 |
| SOCS5 | SPARCL1 | SSBP2 | STARD5 | STK3 | STX7 | SULF1 |
| SYT1 | TBC1D19 | TLR1 | TLR3 | TMEM155 | TMEM59 | TNFRSF11B |
| TNFSF4 | TPD52L1 | TRPC1 | TRPC4 | TTC14 | TTC37 | TXNIP |
| UACA | USP15 | UTY | VCAM1 | VIT | VLDLR | VMP1 |
| WDR19 | WDR63 | WEE1 | XRCC4 | ZMAT1 | ZMYM5 | ZNF184 |
| ZNF277 | ZNF302 | ZNF415 | ZNF484 |  |  |  |
|  | | | | | | |

| **DE-mRNA downregulated in SMC incubated with EVOO-EVs** | | | | | | |
| --- | --- | --- | --- | --- | --- | --- |
| ABCA2 | ABCA3 | ABCB6 | ABCC1 | ABCC3 | ABCD1 | ABHD2 |
| ABL1 | ACE | ACP2 | ADAMTS14 | ADAMTS15 | ADAMTS18 | ADAMTS7 |
| ADAMTSL2 | ADAP1 | ADAT3, SCAMP4 | ADCY9 | AEBP1 | AFAP1 | AGO2 |
| AGRN | AHNAK2 | AKAP2,PALM2,PALM2-AKAP2 | AMOTL1 | ANGPTL4 | ANKRD1 | ANKRD52 |
| ANKRD54 | ANPEP | AP1B1 | AP1M1 | AP2A1 | AP3D1 | APBA2 |
| APCDD1L | APLN | ARHGAP1 | ARHGAP22 | ARHGAP23 | ARHGAP39 | ARHGDIA |
| ARID1A | ARID1B | ARID3A | ASB1 | ATAD3A | ATG2A | ATP13A1 |
| ATP13A2 | ATP6V0A1 | ATP6V0C | AXL | BAG3 | BAHCC1 | BAHD1 |
| BAI2 | BCAM | BCAR1 | BCL9L | BCORL1 | BCR | BEND3P3 |
| BIRC3 | BMP2 | BOP1 | BRD4 | C11orf68 | C14orf80 | C15orf39 |
| C15orf48 | C15orf52 | C17orf97 | C19orf24 | C19orf55 | C1orf233 | C20orf27 |
| C6orf132 | CAD | CALB2 | CAMK2N1 | CAPN15 | CAPN5 | CCDC106 |
| CCDC124 | CCDC85B | CCDC86 | CCDC9 | CCDC94 | CCIN | CCK |
| CCL20 | CCND1 | CCND3 | CCRN4L | CD82 | CD97 | CDC42EP2 |
| CDC42EP4 | CDCP1 | CDH4 | CDT1 | CEBPB | CENPB | CEP170B |
| CERS1,GDF1 | CHERP | CHST2 | CHST3 | CHST7 | CHTF18 | CIC |
| CILP2 | CIZ1 | CKB | CLCN4 | CLDN1 | CLDN11 | CLDN14 |
| CLIP2 | CLPTM1 | CLUH | CMIP | COL13A1 | COL22A1 | COL5A1 |
| COL5A3 | COL7A1 | COQ4 | CORO2B | COTL1 | CREB3L1 | CREB3L2 |
| CREBBP | CRLF1 | CROCC | CSF1 | CSF3 | CSK | CSRNP1 |
| CTDP1 | CXCL1 | CXCL2 | CXCL3 | CXCL5 | CXCL6 | CYGB |
| DAB2IP | DAG1 | DAGLA | DHCR24 | DHCR7 | DHODH | DHX34 |
| DHX37 | DMBT1 | DNER | DNLZ | DNMBP | DNMT1 | DOCK5 |
| DOK3 | DPF3 | DPP3 | DPP9 | DPYSL4 | DRAP1 | DTX2 |
| DUSP4 | DUSP5 | DUSP6 | DYNLL2 | DYRK1B | E2F1 | ECE1 |
| EHD1 | EHD2 | EIF4G1 | EIF5A | ELK1 | ELMSAN1 | EP300 |
| EP400 | EPHA2 | EPN1 | ETV4 | ETV5 | EVC,LOC102723901 | FADS2 |
| FADS3 | FAM101B | FAM129B | FAM163A | FAM167A | FAM171A1 | FAM213B |
| FAM219A | FAM65A | FAM83G | FAM86DP | FARP1 | FASN | FBLN7 |
| FBRS | FBRSL1 | FBXL6,TMEM249 | FGD1 | FGF5 | FGFRL1 | FIZ1 |
| FJX1 | FLNB | FLNC | FNDC1 | FOSL1 | FOSL2 | FOXC2 |
| FOXD1 | FOXJ2 | FOXP4 | FOXRED2 | FPGS | FRMD4A | FSCN1 |
| FTSJ1 | FURIN | GABBR2 | GALNT6 | GAS2L1 | GATA3 | GATA4, LOC101929490 |
| GATAD2A | GDNF | GJD3 | GLI3 | GLTPD1 | GLTSCR1 | GNA11 |
| GPR4 | GPR56 | GPR68 | GPRC5B | GRAMD1A | GRINA | GYS1 |
| HBEGF | HCFC1 | HCN2 | HDAC4 | HDGF | HELZ2 | HERC2 |
| HIPK2 | HK1 | HK2 | HLX | HMGA1 | HMOX1 | HPCAL1 |
| HS6ST3 | HSPA1B | HSPB6 | HSPG2 | HTT | ICAM1 | IER3 |
| IGF1R | IGFBP4 | IGFBP5 | IL17RA | IL1B | IL32 | IL33 |
| IL4R | IL8 | IMPDH1 | INCENP | INTS1 | IPO4 | IQSEC1 |
| IRAK1 | IRF1 | IRX3 | ISM2 | ITFG3 | ITGA10 | ITPR3 |
| ITPRIP | JUND | JUP | KCNC4 | KCNG1 | KDM4B | KDR |
| KHSRP | KIAA0930 | KIAA1549 | KIAA1549L | KIAA1671 | KIF1C | KIF26B |
| KIRREL3 | KLC2 | KLF16 | KLF2 | KLF4 | KLF6 | KLHL21 |
| KSR1 | LAMC2 | LARP1 | LDLR | LDOC1L | LETM1 | LIF |
| LIMK1 | LMNB2 | LMTK2 | LOC100996433 | LOC102723610, RAD54L2 | LOC102724249, SLC7A1 | LOC102724258, SPRED2 |
| LOC102724833, MGAT1 | LOC344887 | LOC90784 | LONP1 | LOXL1 | LOXL4 | LPCAT1 |
| LPCAT4 | LPHN1 | LPPR2 | LPXN | LRFN3 | LRIG1 | LRP1 |
| LRP5 | LRRC15 | LRRC38 | LRRC8A | LSS | LTBP2 | LTBP4 |
| LYNX1 | MAFF | MAP1A | MAP1S | MAP2K2 | MAP2K3 | MAP3K10 |
| MAP7D1 | MAPK8IP1 | MARCH4 | MARK2 | MARVELD1 | MBOAT7 | MCAM |
| MDC1 | MED25 | MEF2D | MEGF8 | MESDC1 | MFSD12 | MFSD2A |
| MGAT5 | MGLL | MGRN1 | MICALL1 | MICALL2 | MIDN | MIER2 |
| MIR137,MIR137HG | MLXIP | MMP17 | MNT | MPRIP | MSTO1 | MT1E |
| MT1G | MT1L | MT1X | MT2A | MTSS1L | MUC13 | MVB12B |
| MYBBP1A | MYO10 | MYO9B | MYPOP | NACC1 | NACC2 | NAV1 |
| NAV2 | NCDN,TFAP2E | NCKAP5L | NCLN | NCOR2 | NCS1 | NDRG4 |
| NEFM | NFIC | NFIX | NFKB2 | NFKBIA | NKD2 | NOC2L |
| NOC4L | NOL6 | NOTCH1 | NOTCH2 | NOTCH3 | NPAS2 | NPDC1 |
| NPLOC4 | NPTX1 | NPTXR | NR1D1 | NR4A1 | NRXN2 | NUAK2 |
| NUBP2 | OAF | OGDH | OLFM2 | OPLAH | ORAI1 | OSBP2 |
| OSGIN1 | PACS1 | PAG1 | PAK4 | PANX2 | PAQR4 | PAQR7 |
| PCNXL3 | PDE4A | PDXP | PEG10 | PELP1 | PER1 | PF4V1 |
| PFAS | PFKFB3 | PFKFB4 | PFKP | PHLDA1 | PHLDA2 | PHRF1 |
| PIEZO1 | PIK3R2 | PIP5K1C | PITPNA | PITPNM2 | PKN1 | PLA2G4C |
| PLEC | PLEKHN1 | PLXNA1 | PLXNA2 | PLXNA4 | PML | PNPLA2 |
| PNPLA6 | POLR1A | POLR2A | POM121 | POM121C | PORCN | POU2F2 |
| PPP5C | PPP6R1 | PPRC1 | PQLC2 | PRDM1 | PREX1 | PRKACA |
| PRKCA | PRKCD | PRKCSH | PRR12 | PRRC2A | PRRC2B | PRRT3 |
| PTGS2 | PTMS | PTPN23 | PTPRF | PTPRS | PVRL1 | PVRL2 |
| PXN | QSOX2 | RAB11FIP5 | RAB36 | RAD23A | RAMP1 | RANGAP1 |
| RAPGEF1 | RASA3 | RAVER1 | RELA | RELB | RERE | REXO1 |
| RFX1 | RFX8 | RHBDF2 | RHOB | RIN1 | RNF126 | RNF187 |
| RNF208 | RNPEPL1 | RPS6KA2 | RPS6KA4 | RPTOR | RRBP1 | RREB1 |
| RRP12 | RRP9 | RRS1 | S1PR2 | SAMD1 | SAMD10 | SART1 |
| SCAF1 | SCAF4 | SCAP | SCARB1 | SCN1B | SCRIB | SEC16A |
| SECTM1 | SEMA3F | SEMA7A | SEPT9 | SERINC2 | SERPINB2 | SETD1B |
| SEZ6L2 | SF3A1 | SF3A2 | SGK223 | SGSH | SH2B3 | SH2D5 |
| SH3BP1 | SH3GL1 | SH3PXD2B | SHB | SHISA5 | SIK3 | SIPA1L3 |
| SKI | SLC16A3 | SLC19A1 | SLC22A23 | SLC25A1 | SLC25A10 | SLC25A23 |
| SLC27A4 | SLC2A1 | SLC2A6 | SLC35F6 | SLC38A5 | SLC39A3 | SLC43A2 |
| SLC52A2 | SLC6A8 | SLC7A5 | SLC8A1 | SLC9A3R2 | SLX4 | SMAP2 |
| SMARCA4 | SMOX | SMTN | SNAPC4 | SOD3 | SOGA1 | SORBS3 |
| SOX12 | SOX17 | SPATA2 | SPEN | SPHK1 | SPON1 | SPRED3 |
| SPRY2 | SPRY4 | SPTBN2 | SRCAP | SREBF2 | SRM | SRXN1 |
| SSC5D | ST3GAL2 | STATH | STC1 | STC2 | STK10 | STXBP1 |
| SUV39H1 | SVIL | SYMPK | SYNJ2 | TACC2 | TAPBP,ZBTB22 | TBC1D10B |
| TBC1D13 | TBC1D24 | TBX3 | TCF19 | TCF3 | TCF7L1 | TCOF1 |
| TENM4 | TET3 | TFAP2A | TFAP4 | TFE3 | TFPI2 | TGFA |
| TGFB1 | TGFBR3L | TGM2 | THBD | THEM6 | THOP1 | TICAM1 |
| TIMP3 | TJAP1 | TLE3 | TMEM104 | TMEM132A | TMEM158 | TMEM171 |
| TMEM178B | TMEM184B | TMEM201 | TNC | TNFAIP3 | TNFRSF11A | TNIP1 |
| TNKS1BP1 | TNRC18 | TOM1L2 | TOMM40 | TOR4A | TP53 | TRAF3 |
| TRERF1 | TRIM16 | TRIM25 | TRIM28 | TRIM56 | TRIM62 | TRIM8 |
| TSC22D2 | TSC22D4 | TSKU | TSPAN14 | TTLL12 | TTYH3 | TUBB3 |
| TUBB4B | TWIST2 | UBASH3B | UBE2J2 | UBE2M | UBE2O | UHRF1 |
| UPF1 | VARS | VAV2 | VIPR1 | VPS4A | WDR81 | WFS1 |
| WIZ | WNK4 | WWC1 | WWC3 | XYLT1 | YTHDF1 | ZBTB45 |
| ZBTB7A | ZC3H12A | ZC3H18 | ZC3H4 | ZC3H7B | ZCCHC14 | ZDHHC8 |
| ZFHX3 | ZFPM1 | ZFYVE28 | ZMIZ1 | ZMIZ2 | ZNF142 | ZNF213 |
| ZNF341 | ZNF469 | ZNF574 | ZNF598 | ZNF618 | ZNF628 | ZNF629 |
| ZNF703 | ZNF710 | ZNF777 | ZNF837 | ZNF865 | ZSWIM4 | ZYX |

Supplementary Table 4. EVOO-mRNAs and SO-mRNAs involved in the 10 more significant GO:BP analyzed.

| **GO:BP** |  | **EVOO-mRNAs** | **SO-mRNAs** |
| --- | --- | --- | --- |
| **Response to endogenous stimulus (GO:0009719)** |  | AKAP6, AKR1C3, ANG, ANXA3, ASNS, BCHE, EDNRB, ELAPOR2, FGF7, FIBIN, HNMT, ITGA4, LRRK2, MGARP, NOX4, OGT, OSBPL8, PDGFD, PRCP, PTK2B, SESN3, SMYD3, SOCS5, SULF1, TNFRSF11B, TNFSF4, TXNIP, USP15, VCAM1. | ABCA2, ABCA3, ABCC1, ABHD2, ABL1, ADAMTS7, ADAMTSL2, ADCY9, ANKRD1, APLN, ARID1A, ARID1B, ATP6V0A1, ATP6V0C, BCAR1, BCL9L, BMP2, CAD, CCND1, CCND3, CEBPB, CLDN1, CREB3L1, CREBBP, CSK, CXCL8, DAB2IP, DAG1, DHCR24, DNMT1, E2F1, EHD1, ELK1, EP300, FAM83G, FGF5, FGFRL1, FOSL1, FOXC2, FOXD1, FURIN, GAS2L1, GATA3, GATA4, GLI3, GNA11, HCN2, HDAC4, HIPK2, HMOX1, ICAM1, IGF1R, IGFBP5, IL1B, IL4R, JUND, JUP, KLF16, KLF2, KLF4, LARP1, LDLR, LONP1, LPXN, LRP1, LRP5, LTBP2, LTBP4, MEGF8, MICALL1, MTSS2, NCOR2, NOTCH1, NOTCH2, NR1D1, NR4A1, PAQR7, PDXP, PEG10, PELP1, PER1, PIK3R2, PML, POLR2A, PPP5C, PRKACA, PRKCD, PTGS2, PXN, RAMP1, RANGAP1, RAPGEF1, RELA, RPTOR, SCAP, SKI, SLC2A1, SLC8A1, SLX4, SMARCA4, SOGA1, SPRED2, SPRED3, SPRY2, SPRY4, SREBF2, STC1, STC2, TFAP4, TGFB1, TGFBR3L, TICAM1, TIMP3, TNC, TP53, TRERF1, TRIM16, TRIM25, UBE2O, ZBTB7A, ZFHX3, ZMIZ1, ZNF703, ZYX. |
|  |  |  |  |
|  |  |  |  |
|  |  |  |  |
|  |  |  |  |
|  |  |  |  |
|  |  |  |  |
|  |  |  |  |
|  |  |  |  |
|  |  |  |  |
|  |  |  |  |
| **Positive regulation of cellular biosynthetic process (GO:0031328)** |  | CAND1, EIF3E, F2R, FBXO4, FGF7, FXR1, GTF2A1L, HCFC2, HLTF, ID4, LDB2, LUM, MEIS1, NFYB, NIBAN1, NOX4, OGT, OSR2, PKIB, PLSCR1, PTK2B, SLC40A1, SMARCA1, SMYD3, SSBP2, TLR1, TLR3. | ABCA2, ABCD1, ABL1, AGO2, AGRN, ANKRD1, APLN, ARID1A, ARID1B, ARID3A, BCL9L, BICRA, BMP2, BRD4, CDT1, CEBPB, CHTF18, CREB3L1, CREB3L2, CREBBP, CSF3, CSRNP1, CXCL8, DAB2IP, DPF3, DRAP1, DYRK1B, E2F1, EIF4G1, EIF5A, ELK1, EP300, ETV4, ETV5, FOSL1, FOSL2, FOXC2, FOXD1, FOXJ2, GATA3, GATA4, GDNF, GLI3, HCFC1, HDAC4, HELZ2, HIPK2, HMGA1, HMOX1, ICAM1, IL1B, IL33, IRF1, JUND, JUP, KLF2, KLF4, KLF6, LARP1, LDLR, LIF, LRP5, MAFF, MAP2K2, MAP2K3, MED25, MEF2D, MFSD2A, MLXIP, NFIC, NFIX, NFKB2, NFKBIA, NIBAN2, NOTCH1, NOTCH3, NPAS2, NR1D1, NR4A1, PELP1, PER1, PIK3R2, PKN1, PML, POU2F2, PPRC1, PRKCD, PTGS2, RAMP1, RELA, RELB, RERE, RNF187, RPS6KA4, RPTOR, RREB1, SCAP, SCARB1, SKI, SMARCA4, SOX12, SOX17, SRCAP, SREBF2, TBX3, TCF3, TCF7L1, TET3, TFAP2A, TFAP4, TFE3, TGFB1, TICAM1, TNIP1, TP53, TRERF1, TRIM16, TRIM28, TRIM56, UHRF1, WIZ, WWC1, YTHDF1, ZC3H12A, ZFHX3, ZFPM1, ZMIZ1, ZMIZ2, ZNF341. |
|  |  |  |  |
|  |  |  |  |
|  |  |  |  |
|  |  |  |  |
|  |  |  |  |
|  |  |  |  |
|  |  |  |  |
|  |  |  |  |
|  |  |  |  |
|  |  |  |  |
| **Regulation of intracellular signal transduction (GO:1902531)** |  | A2M, AKAP6, AKR1C3, ARHGAP20, ARRDC3, CARD16, CD14, DDX60, F2R, FGF7, GPNMB, GUCY1A2, IRAK4, LRRK2, MAPK10, NOX4, OGT, OSBPL8, PDE5A, PDGFD, PLK2, PTK2B, ROR1, SESN3, STK3, TLR3, TPD52L1, UACA, USP15 | ABL1, ADGRG1, ANKRD1, ANKRD54, ARHGAP1, ARHGAP22, ARHGAP23, ARHGAP39, ARHGDIA, AXL, BCR, BIRC3, BMP2, BOP1, BRD4, CAMK2N1, CCL20, CHERP, CREB3L1, CSF1, CSF3, CSK, DAB2IP, DAG1, DUSP4, DUSP5, DUSP6, EP300, EPHA2, FGD1, FGF5, GATA3, GATA4, GPR4, GPRC5B, GRINA, HBEGF, HIPK2, HMOX1, HSPA1B, HTT, ICAM1, IER3, IGF1R, IGFBP4, IGFBP5, IL1B, IQSEC1, IRAK1, KDR, KLF4, KSR1, LIF, LRP1, MAP2K2, MAP2K3, MAP3K10, MAPK8IP1, MGRN1, MIR137, MYO9B, NACC2, NDRG4, NFKBIA, NOC2L, NOTCH1, NOTCH2, NPLOC4, NR1D1, PDE4A, PER1, PIK3R2, PIP5K1C, PKN1, PML, PPP5C, PREX1, PRKACA, PRKCA, PRKCD, PTGS2, RAPGEF1, RASA3, RELA, RHOB, RPTOR, RRS1, S1PR2, SECTM1, SEMA7A, SH2B3, SH3BP1, SHISA5, SIK3, SIPA1L3, SORBS3, SPHK1, SPRED2, SPRED3, SPRY2, SPRY4, TGFA, TGFB1, TGM2, TICAM1, TIMP3, TNFAIP3, TNFRSF11A, TNIP1, TP53, TRAF3, TRIM25, TRIM62, TRIM8, VAV2, WFS1, WWC1, WWC3, YJU2, ZC3H12A. |
|  |  |  |  |
|  |  |  |  |
|  |  |  |  |
|  |  |  |  |
|  |  |  |  |
|  |  |  |  |
|  |  |  |  |
|  |  |  |  |
|  |  |  |  |
| **Positive regulation of multicellular organismal process (GO:0051240)** |  | AKAP6, ANXA3, CD14, DDX60, DECR1, EDNRB, F2R, FGF7, LRRK2, LUM, LY96, N4BP2L2, OGT, OMA1, OSR2, PDE5A, PDGFD, PLK2, PTK2B, RNF112, ROR1, SERPINB7, SOCS5, SULF1, SYT1, TLR1, TLR3, TNFSF4, USP15, VLDLR | ABL1, ADGRB2, ADGRL1, AGO2, AGRN, AMOTL1, ANGPTL4, ANKRD1, ANKRD54, AP2A1, AP3D1, APLN, ARHGDIA, AXL, BCAR1, BCL9L, BIRC3, BMP2, CCK, CCND1, CDH4, CEBPB, CREB3L2, CREBBP, CSF1, CSF3, CXCL8, DAB2IP, DAG1, DHX37, DOCK5, E2F1, EIF4G1, EP300, ETV4, ETV5, FOXC2, FOXD1, FURIN, GATA3, GATA4, GDNF, GLI3, GPR68, GPRC5B, HBEGF, HDAC4, HIPK2, HK2, HLX, HMOX1, HSPA1B, HSPB6, ICAM1, IGF1R, IL17RA, IL1B, IL33, IL4R, IQSEC1, IRAK1, IRF1, IRX3, JUND, JUP, KDR, KLF4, LIF, LIMK1, LRP1, LRP5, MAP2K3, MARK2, MEGF8, MIR137, NDRG4, NFKB2, NIBAN2, NOTCH1, NOTCH2, NPAS2, NPLOC4, PAK4, PLXNA1, PLXNA2, PLXNA4, POU2F2, PRKCA, PTGS2, RAPGEF1, RELA, RHOB, RREB1, SART1, SCARB1, SCN1B, SCRIB, SEMA7A, SF3A2, SH3PXD2B, SLC7A5, SLC8A1, SOX12, SPEN, SPHK1, TCF3, TENM4, TFAP2A, TGFB1, THBD, TICAM1, TNFAIP3, TNFRSF11A, TRIM16, ZC3H12A, ZFPM1, ZMIZ1, ZNF703 |
|  |  |  |  |
|  |  |  |  |
|  |  |  |  |
|  |  |  |  |
|  |  |  |  |
|  |  |  |  |
|  |  |  |  |
|  |  |  |  |
|  |  |  |  |
| **Regulation of phosphorus metabolic process (GO:0051174)** |  | ANG, CCNG1, EDNRB, F2R, FGF7, FXR1, GPNMB, KCTD20, LDB2, LGALS3, LIMCH1, LRRK2, MAPK10, NDUFS4, NIBAN1, NOX4, NUP37, OGT, OSBPL8, PDE5A, PDGFD, PKIB, PLCL1, PTK2B, ROR1, SMYD3, SOCS5, STK3, TLR3, TPD52L1, VLDLR | ABCA2, ABCD1, ABL1, ACE, ADCY9, ANKRD54, APLN, AXL, BMP2, BRD4, CAMK2N1, CCK, CCL20, CCND1, CCND3, CRLF1, CSF1, CSF3, CSK, DAB2IP, DAG1, DUSP4, DUSP5, DUSP6, EIF4G1, EP300, EPHA2, FARP1, FGF5, FIZ1, GATA4, GPRC5B, HBEGF, HDAC4, HIPK2, HTT, ICAM1, IGF1R, IGFBP4, IL1B, INCENP, IRAK1, ITPRIP, KDR, KLF4, KSR1, LDLR, LIF, LMTK2, LPCAT1, LRP1, LRP5, MAP2K2, MAP2K3, MAP3K10, MAPK8IP1, MARK2, MGAT5, MIDN, MIR137, NDRG4, NOTCH1, NOTCH2, PAK4, PER1, PFKFB3, PFKFB4, PIK3R2, PKN1, PML, POM121, POM121C, PPP5C, PPP6R1, PRKACA, PRKCA, PRKCD, PTGS2, RAPGEF1, RPS6KA4, RPTOR, S1PR2, SCARB1, SEMA7A, SH2B3, SLC25A23, SLC2A6, SLC8A1, SORBS3, SPHK1, SPRED2, SPRED3, SPRY2, SPRY4, SYMPK, TFAP4, TGFA, TGFB1, TIMP3, TNFAIP3, TNFRSF11A, TNIP1, TNKS1BP1, TP53, TRAF3, UBASH3B, VAV2, WDR81, WWC1, ZBTB7A, ZC3H12A, ZFYVE28 |
|  |  |  |  |
|  |  |  |  |
|  |  |  |  |
|  |  |  |  |
|  |  |  |  |
|  |  |  |  |
|  |  |  |  |
|  |  |  |  |
|  |  |  |  |
| **Cell motility (GO:0048870)** |  | ANG, ANXA3, EDNRB, EFHC1, F2R, FAP, FGF7, GPC6, GPNMB, IGSF10, IRAK4, ITGA4, JCHAIN, LAMA2, LDB2, LGALS3, LIMCH1, LRRK2, MCU, MTUS1, MYH10, NOX4, OSBPL8, PDGFD, PLK2, PRCP, PROS1, PTK2B, SULF1, VCAM1, WDR63 | ABCC1, ABHD2, ABL1, ACE, ADGRG1, AGO2, AMOTL1, AXL, BCAR1, BCR, BMP2, CCK, CCL20, CLDN1, COL5A1, CSF1, CXCL1, CXCL2, CXCL3, CXCL5, CXCL6, CXCL8, CYGB, DAB2IP, DAG1, DNER, DOCK5, EPHA2, FGF5, FOXC2, FSCN1, GATA3, GDNF, GLI3, HBEGF, HDAC4, HMOX1, ICAM1, IGF1R, IGFBP5, IL17RA, IL1B, IL33, IQSEC1, JUP, KDR, KIRREL3, KLF4, LAMC2, LPXN, LRP1, LRP5, LRRC15, MAP2K3, MARK2, MBOAT7, MCAM, MEGF8, MGAT5, MIR137, NAV1, NDRG4, NOTCH1, NR4A1, OSGIN1, PAK4, PF4V1, PHLDA2, PIK3R2, PIP5K1C, PKN1, PLXNA1, PLXNA2, PLXNA4, PML, PREX1, PRKCA, PRKCD, PTGS2, PTPN23, PTPRF, PXN, RERE, RHOB, RIPOR1, RREB1, SCARB1, SCRIB, SEMA3F, SEMA7A, SH3BP1, SKI, SLC16A3, SLC7A5, SLC8A1, SOX17, SPHK1, SPRY2, STC1, STK10, TGFB1, TGFBR3L, THBD, TMEM201, TNFAIP3, TNFRSF11A, TWIST2, VAV2, WWC1, ZC3H12A, ZMIZ1, ZNF703 |
|  |  |  |  |
|  |  |  |  |
|  |  |  |  |
|  |  |  |  |
|  |  |  |  |
|  |  |  |  |
|  |  |  |  |
|  |  |  |  |
|  |  |  |  |
| **Regulation of cell population proliferation (GO:0042127)** |  | AKR1C3, ANG, BCHE, CALCRL, EDNRB, F2R, FAP, FBXO4, FGF7, FRK, GPNMB, ID4, IFT52, INTU, IRAK4, ITGA4, LGALS3, LRRK2, MEIS1, N4BP2L2, NDUFS4, NOX4, OSR2, PDE1A, PDE5A, PDGFD, PTK2B, RARRES1, SERPINB7, STK3, SULF1, TNFSF4, TXNIP, VCAM1 | ABL1, ACE, ADGRG1, APLN, BMP2, CAMK2N1, CCK, CCND1, CCND3, CEBPB, CHERP, CLDN1, CRLF1, CSF1, CSF3, CSK, CXCL1, CXCL5, CXCL8, DAB2IP, DHCR24, DHCR7, DNMT1, E2F1, EIF5A, ETV5, FGF5, FGFRL1, FOSL1, FOSL2, FOXJ2, GATA3, GDNF, GLI3, HBEGF, HDAC4, HIPK2, HLX, HMGA1, HMOX1, HSPA1B, IGF1R, IGFBP5, IL1B, IL33, IL4R, IRAK1, IRF1, JUND, JUP, KDR, KLF4, KSR1, LAMC2, LIF, LRP5, MAPK8IP1, MED25, MEF2D, MIR137, MNT, NACC1, NACC2, NDRG4, NFKBIA, NIBAN2, NOTCH1, NOTCH2, NOTCH3, NR1D1, NR4A1, OSGIN1, PHLDA2, PKN1, PML, PRDM1, PRKCA, PTGS2, RAPGEF1, RELA, RNF126, RNF187, RPS6KA2, RPTOR, RREB1, S1PR2, SCRIB, SH2B3, SKI, SLC35F6, SLC7A1, SOX17, SPHK1, SPRY2, TBX3, TFAP2A, TFAP4, TGFA, TGFB1, TGM2, TICAM1, TNC, TNFAIP3, TNFRSF11A, TP53, UHRF1, VIPR1, ZMIZ1, ZNF703 |
|  |  |  |  |
|  |  |  |  |
|  |  |  |  |
|  |  |  |  |
|  |  |  |  |
|  |  |  |  |
|  |  |  |  |
|  |  |  |  |
|  |  |  |  |
| **Response to oxygen-containing compound (GO:1901700)** |  | AKAP6, AKR1C3, ASNS, BCHE, CALCRL, CARD16, CD14, EDNRB, ESD, F2R, FIBIN, FMO1, HNMT, ITGA4, LRRK2, LY96, MAPK10, MGARP, NDUFS4, NOX4, OGT, OSBPL8, PDGFD, PLSCR4, PTK2B, RNF112, SESN3, SLC38A2, SMYD3, TNFSF4, TXNIP, VCAM1, ZNF277 | ABCA2, ABCC1, ABHD2, ABL1, ADCY9, AGRN, ANKRD1, ARID1B, ATP6V0A1, ATP6V0C, AXL, BCAR1, BCR, CAD, CCND1, CCND3, CDT1, CEBPB, CERS1, CLDN1, CSF3, CSK, CXCL1, CXCL2, CXCL3, CXCL5, CXCL6, CXCL8, DAB2IP, DAG1, DHODH, DNMT1, E2F1, ELK1, FOSL1, FOXC2, GATA3, GATA4, GJD3, GNA11, GPR68, GRAMD1A, HCN2, HMOX1, ICAM1, IGF1R, IGFBP5, IL1B, IRAK1, JUND, JUP, KLF16, KLF2, KLF4, LARP1, LDLR, LOXL1, LRP1, LRP5, NCOR2, NFKB2, NFKBIA, NOCT, NOTCH1, NR1D1, NR4A1, PDXP, PER1, PF4V1, PIK3R2, PPP5C, PRKACA, PRKCA, PRKCD, PTGS2, PXN, RAB11FIP5, RANGAP1, RAPGEF1, RELA, RHOB, RPTOR, SCAP, SCARB1, SLC2A1, SLC8A1, SMARCA4, SOD3, SOGA1, SPHK1, STC1, STC2, STXBP1, TFAP4, TGFB1, THBD, TICAM1, TNC, TNFAIP3, TNFRSF11A, TP53, TRERF1, TRIM16, TRIM25, UPF1, ZC3H12A, ZNF703 |
|  |  |  |  |
|  |  |  |  |
|  |  |  |  |
|  |  |  |  |
|  |  |  |  |
|  |  |  |  |
|  |  |  |  |
|  |  |  |  |
|  |  |  |  |
| **Biological adhesion (GO:0022610)** |  | CDH8, COL14A1, CSTA, CTNNAL1, EMB, FAP, FMN1, GPC6, GPNMB, ITGA4, LAMA2, LGALS3, LIMCH1, MYH10, PCDH18, PDE5A, PTK2B, SOCS5, SPARCL1, TNFSF4, VCAM1, VIT, VMP1 | ABL1, ADAMTS18, ADGRE5, ADGRG1, ADGRL1, AP3D1, ARHGDIA, AXL, BCAM, BCAR1, BCR, BMP2, CDH4, CEBPB, CLDN1, CLDN11, CLDN14, COL13A1, COL5A1, COL5A3, COL7A1, CORO2B, CSF1, CSK, CXCL8, DAG1, DOCK5, EPHA2, FBLN7, FGFRL1, FOXC2, GATA3, GLI3, GPR4, HLX, ICAM1, IL1B, IL32, IL4R, IRAK1, IRF1, ITGA10, JUP, KDR, KIF26B, KIRREL3, KLF4, LAMC2, LIF, LPXN, LRFN3, LRP1, LRRC15, MCAM, MICALL2, MYO10, NECTIN1, NECTIN2, NOTCH1, NRXN2, PAG1, PAK4, PIEZO1, PIP5K1C, PLXNA1, PLXNA2, PLXNA4, PML, PREX1, PRKCA, PRKCD, PTPN23, PTPRF, PTPRS, PXN, RAPGEF1, RELA, RHOB, RREB1, SART1, SCARB1, SCN1B, SCRIB, SH2B3, SLC7A1, SORBS3, SOX12, SPON1, STK10, STXBP1, SYMPK, TENM4, TFE3, TGFB1, TGM2, TNC, TNIP1, UBASH3B, ZC3H12A, ZFHX3, ZMIZ1, ZNF703, ZYX |
|  |  |  |  |
|  |  |  |  |
|  |  |  |  |
|  |  |  |  |
|  |  |  |  |
|  |  |  |  |
|  |  |  |  |
|  |  |  |  |
| **Positive regulation of nucleobase-containing compound metabolic process (GO:0045935)** |  | ANXA3, CAND1, F2R, FBXO4, FGF7, GTF2A1L, HCFC2, HLTF, HNRNPLL, ID4, LDB2, LUM, MEIS1, NFYB, NOX4, OGT, OSR2, PKIB, PLSCR1, PTK2B, SLC38A2, SLC40A1, SMARCA1, SMYD3, SSBP2, TLR3, TNFSF4 | ABL1, AGO2, AGRN, ANKRD1, APLN, ARID1A, ARID1B, ARID3A, BCL9L, BICRA, BMP2, BRD4, CEBPB, CHTF18, CREB3L1, CREB3L2, CREBBP, CSF3, CSRNP1, DAB2IP, DPF3, DRAP1, DYRK1B, E2F1, ELK1, EP300, ETV4, ETV5, FOSL1, FOSL2, FOXC2, FOXD1, FOXJ2, GATA3, GATA4, GDNF, GLI3, HCFC1, HDAC4, HELZ2, HIPK2, HMGA1, IL1B, IL33, IRF1, JUND, JUP, KHSRP, KLF2, KLF4, KLF6, LIF, LRP5, MAFF, MAP2K2, MAP2K3, MED25, MEF2D, MLXIP, NFIC, NFIX, NFKB2, NFKBIA, NIBAN2, NOTCH1, NOTCH3, NPAS2, NR1D1, NR4A1, PELP1, PER1, PFKFB3, PFKFB4, PIK3R2, PKN1, PLEKHN1, PML, POLR2A, POU2F2, PPRC1, PRKCD, RELA, RELB, RERE, RNF187, RPS6KA4, RPTOR, RREB1, SCAP, SKI, SLX4, SMARCA4, SOX12, SOX17, SRCAP, SREBF2, TBX3, TCF3, TCF7L1, TET3, TFAP2A, TFAP4, TFE3, TGFB1, TNIP1, TP53, TRERF1, TRIM16, TRIM28, TRIM56, UHRF1, UPF1, WWC1, YTHDF1, ZC3H12A, ZFHX3, ZFPM1, ZMIZ1, ZMIZ2, ZNF341 |
|  |  |  |  |
|  |  |  |  |
|  |  |  |  |
|  |  |  |  |
|  |  |  |  |
|  |  |  |  |
|  |  |  |  |
|  |  |  |  |
|  |  |  |  |

Supplementary Table 5. EVOO-mRNAs and SO-mRNAs involved in the 10 more significant KEGG pathways analyzed.

| **KEGG pathway** | **EVOO-mRNAs** | **SO-mRNAs** |
| --- | --- | --- |
| **Focal adhesión (hsa04510)** | ITGA4, LAMA2, MAPK10, PDGFD | BCAR1, BIRC3, CCND1, CCND3, COL5A1, COL5A3, ELK1, FLNB, FLNC, IGF1R, ITGA10, KDR, LAMC2, PAK4, PIK3R2, PIP5K1C, PRKCA, PXN, RAPGEF1, TNC, VAV2, ZYX |
| **Pathways in cancer (hsa05200)** | FGF7, LAMA2, MAPK10 | ABL1, BCR, BIRC3, BMP2, CCND1, CREBBP, CXCL8, E2F1, EP300, FGF5, GLI3, IGF1R, JUP, LAMC2, MAP2K2, NFKB2, NFKBIA, PIK3R2, PML, PRKCA, PTGS2, RELA, SLC2A1, TCF7L1, TGFA, TGFB1, TP53, TRAF3 |
| **Prostate cancer (hsa05215)** | PDGFD | CCND1, CREB3L1, CREB3L2, CREBBP, E2F1, EP300, IGF1R, MAP2K2, NFKBIA, PIK3R2, RELA, TCF7L1, TGFA, TP53 |
| **Endocytosis (hsa04144)** | EEA1, F2R | AP2A1, EHD1, EHD2, EPN1, HSPA1B, IGF1R, IQSEC1, KDR, LDLR, MVB12B, PIP5K1C, RAB11FIP5, SH3GL1, SMAP2, VPS4A |
| **Regulation of actin cytoskeleton (hsa04810)** | CD14, F2R, FGF7, ITGA4, MYH10, PDGFD | BCAR1, CSK, FGD1, FGF5, ITGA10, LIMK1, MAP2K2, PAK4, PIK3R2, PIP5K1C, PXN, VAV2 |
| **MAPK signaling pathway (hsa04010)** | CACNB4, CD14, FGF7, MAPK10, STK3 | DUSP4, DUSP5, DUSP6, ELK1, FGF5, FLNB, FLNC, HSPA1B, IL1B, JUND, MAP2K2, MAP2K3, MAPK8IP1, NFKB2, NR4A1, PPP5C, PRKACA, PRKCA, RELA, RELB, RPS6KA2, RPS6KA4, TGFB1, TP53 |
| **Toll-like receptor signaling pathway (hsa04620)** | CD14, CTSK, IRAK4, LY96, MAPK10, TLR1, TLR3 | CXCL8, IL1B, IRAK1, MAP2K2, MAP2K3, NFKBIA, PIK3R2, RELA, TICAM1, TRAF3 |
| **Neurotrophin signaling pathway (hsa04722)** | IRAK4, MAPK10 | ABL1, ARHGDIA, CSK, IRAK1, MAP2K2, NFKBIA, PIK3R2, PRKCD, RAPGEF1, RELA, RPS6KA2, RPS6KA4, SH2B3, TP53 |
| **Chemokine signaling pathway (hsa04062)** | PTK2B | ADCY9, BCAR1, CCL20, CSK, CXCL1, CXCL2, CXCL3, CXCL5, CXCL6, CXCL8, NFKBIA, PF4V1, PIK3R2, PREX1, PRKACA, PRKCD, PXN, RELA, VAV2 |
| **Wnt signaling pathway (hsa04310)** | AC023512.1, MAPK10 | CCND1, CCND3, CREBBP, EP300, FOSL1, LRP5, NKD2, PORCN, PRKACA, PRKCA, SOX17, TCF7L1, TP53 |

**Supplementary Table 6.** Interactions found in our study which were experimentally observed according to different sources between miRNAs found in extracellular vesicles secreted from HUVECs cells and their target genes in smooth muscle cells.

| **miRNA** | **Target gene** | **Source** | **Reference** |
| --- | --- | --- | --- |
| **Upregulated miRNA in EVOO-EVs -- Upregulated EVOO-mRNAs in SMC** | | | |
| hsa-miR-30b-5p | TMEM59 | TarBase | * |
|  | SLC38A2 | TarBase | 1-4 |
| hsa-miR-16-5p | OGT | Ingenuity Expert Findings | 5,6 |
|  | OMA1 | miRecords | 2 |
|  | FGF7 | Ingenuity Expert Findings,  TargetScan Human | * |
| **Upregulated miRNA in EVOO-EVs -- Downregulated EVOO-mRNAs in SMC** | | | |
| hsa-miR-27b-3p | SRM | miRecords | * |
|  | NOTCH1 | TarBase | 7 |
| hsa-let-7g-5p | ADAMTS14 | Ingenuity Expert Findings,  TargetScan Human | * |
|  | PTGS2 | TarBase | 8 |
|  | FADS2 | TarBase | * |
|  | PRRC2A | TarBase | 4 |
|  | DOCK5 | TarBase | * |
|  | UHRF1 | TarBase | * |
| hsa-miR-23a-3p | NOTCH1 | TarBase | 9 |
| hsa-miR-16-5p | SLC38A5 | TarBase | 4,10 |
|  | SLC16A3 | TarBase | 4,11 |
|  | PTGS2 | TarBase | 12,13 |
|  | HSPA1B | TarBase,  TargetScan Human,  miRecords | 3,5,6 |
|  | ARHGDIA | TarBase,  TargetScan Human | 4,10,13,14 |
|  | IGF1R | Ingenuity Expert Findings,  TargetScan Human | 15-17 |
|  | NOTCH2 | TarBase,  TargetScan Human | 18 |
|  | ACP2 | TarBase,  TargetScan Human | 19,20 |
| hsa-miR-30b-5p | LMNB2 | TarBase | 5,19,21 |
|  | AP2A1 | TarBase,  TargetScan Human | 19,22 |
|  | ADAMTS14 | Ingenuity Expert Findings | * |
| hsa-miR-20a-5p | IL8 | TargetScan Human,  miRecords | 23 |
|  | E2F1 | Ingenuity Expert Findings,  TarBase,  TargetScan Human,  miRecords | 24-29 |
| hsa-miR-148b-3p | DNMT1 | TargetScan Human,  miRecords | 30,31 |
| hsa-miR-216a-3p | LDLR | TargetScan Human,  miRecords | 32 |
| **Downregulated miRNA in EVOO-EVs -- Downregulated EVOO-mRNAs in SMC** | | | |
| hsa-miR-143-3p | SOD3 | Ingenuity Expert Findings,  TargetScan Human | 33-35 |

miRNA in EVOO-EVs: differentially expressed-miRNAs in extracellular vesicle secreted by HUVECs incubated with the TRLs obtained 3 hours after an HF meal with EVOO.

EVOO-mRNAs in SMC: differentially expressed-mRNAs in the smooth mucle cells incubated with the EVOO-EVs.

* No references were found for these miRNA:mRNA pairs, although these interactions were experimentally observed according to the different databases integrated in IPA software (miRecords, Tarbase and TargetScan Human).

**REFERENCES FOR SUPPLEMENTARY TABLE 6**

1. Berti FCB, Mathias C, Garcia LE, Gradia DF, de Araújo-Souza PS, Cipolla GA, de Oliveira JC, Malheiros D. Comprehensive analysis of ceRNA networks in HPV16- and HPV18-mediated cervical cancers reveals XIST as a pivotal competing endogenous RNA. Biochim Biophys Acta Mol Basis Dis. 2021;1867:166172.
2. Xue Y, Ouyang K, Huang J, Zhou Y, Ouyang H, Li H, Wang G, Wu Q, Wei C, Bi Y, Jiang L, Cai Z, Sun H, Zhang K, Zhang Y, Chen J, Fu XD. Direct conversion of fibroblasts to neurons by reprogramming PTB-regulated microRNA circuits. Cell. 2013;152:82-96.
3. Boudreau RL, Jiang P, Gilmore BL, Spengler RM, Tirabassi R, Nelson JA, Ross CA, Xing Y, Davidson BL. Transcriptome-wide discovery of microRNA binding sites in human brain. Neuron. 2014;81:294-305.
4. Balakrishnan I, Yang X, Brown J, Ramakrishnan A, Torok-Storb B, Kabos P, Hesselberth JR, Pillai MM. Genome-wide analysis of miRNA-mRNA interactions in marrow stromal cells. Stem Cells. 2014;32:662-73.
5. Hafner M, Landthaler M, Burger L, Khorshid M, Hausser J, Berninger P, Rothballer A, Ascano M Jr, Jungkamp AC, Munschauer M, Ulrich A, Wardle GS, Dewell S, Zavolan M, Tuschl T. Transcriptome-wide identification of RNA-binding protein and microRNA target sites by PAR-CLIP. Cell. 2010;141:129-41.
6. Karginov FV, Hannon GJ. Remodeling of Ago2-mRNA interactions upon cellular stress reflects miRNA complementarity and correlates with altered translation rates. Genes Dev. 2013;27:1624-32.
7. Fukuda Y, Kawasaki H, Taira K. Exploration of human miRNA target genes in neuronal differentiation. Nucleic Acids Symp Ser (Oxf). 2005;49:341-2.
8. Kim HG, Jung GY, Park SB, Cho YJ, Han M. Assessment of the effects of prostaglandins on myometrial and leiomyoma cells in vitro through microRNA profiling. Mol Med Rep. 2018;18:2499-2505.
9. Chen YC, Lee CP, Hsiao CC, Hsu PY, Wang TY, Wu CC, Chao TY, Leung SY, Chang YP, Lin MC. MicroRNA-23a-3p Down-Regulation in Active Pulmonary Tuberculosis Patients with High Bacterial Burden Inhibits Mononuclear Cell Function and Phagocytosis through TLR4/TNF-α/TGF-β1/IL-10 Signaling via Targeting IRF1/SP1. Int J Mol Sci. 2020;21:8587.
10. Selbach M, Schwanhäusser B, Thierfelder N, Fang Z, Khanin R, Rajewsky N. Widespread changes in protein synthesis induced by microRNAs. Nature. 2008;455:58-63.
11. Whisnant AW, Bogerd HP, Flores O, Ho P, Powers JG, Sharova N, Stevenson M, Chen CH, Cullen BR. In-depth analysis of the interaction of HIV-1 with cellular microRNA biogenesis and effector mechanisms. mBio. 2013;4:e000193.
12. Shen Z, Lu J, Wei J, Zhao J, Wang M, Wang M, Shen X, Lü X, Zhou B, Zhao Y, Fu G. Investigation of the underlying hub genes and mechanisms of reperfusion injury in patients undergoing coronary artery bypass graft surgery by integrated bioinformatic analyses. Ann Transl Med. 2019;7:664.
13. Nelson PT, Wang WX, Mao G, Wilfred BR, Xie K, Jennings MH, Gao Z, Wang X. Specific sequence determinants of miR-15/107 microRNA gene group targets. Nucleic Acids Res. 2011;39:8163-72.
14. Yan X, Liang H, Deng T, Zhu K, Zhang S, Wang N, Jiang X, Wang X, Liu R, Zen K, Zhang CY, Ba Y, Chen X. The identification of novel targets of miR-16 and characterization of their biological functions in cancer cells. Mol Cancer. 2013;12:92.
15. Yin K, Cui Y, Sun T, Qi X, Zhang Y, Lin H. Antagonistic effect of selenium on lead-induced neutrophil apoptosis in chickens via miR-16-5p targeting of PiK3R1 and IGF1R. Chemosphere. 2020;246:125794.
16. Zhang H, Bian C, Tu S, Yin F, Guo P, Zhang J, Song X, Liu Q, Chen C, Han Y. Integrated analysis of lncRNA-miRNA-mRNA ceRNA network in human aortic dissection. BMC Genomics. 2021;22:724.
17. Chen L, Wang Q, Wang GD, Wang HS, Huang Y, Liu XM, Cai XH. miR-16 inhibits cell proliferation by targeting IGF1R and the Raf1-MEK1/2-ERK1/2 pathway in osteosarcoma. FEBS Lett. 2013;587:1366-72.
18. Yao Q, Xing Y, Wang Z, Liang J, Lin Q, Huang M, Chen Y, Lin B, Xu X, Chen W. MiR-16-5p suppresses myofibroblast activation in systemic sclerosis by inhibiting NOTCH signaling. Aging (Albany NY). 2020;13:2640-2654.
19. Kishore S, Jaskiewicz L, Burger L, Hausser J, Khorshid M, Zavolan M. A quantitative analysis of CLIP methods for identifying binding sites of RNA-binding proteins. Nat Methods. 2011;8:559-64.
20. Skalsky RL, Corcoran DL, Gottwein E, Frank CL, Kang D, Hafner M, Nusbaum JD, Feederle R, Delecluse HJ, Luftig MA, Tuschl T, Ohler U, Cullen BR. The viral and cellular microRNA targetome in lymphoblastoid cell lines. PLoS Pathog. 2012;8:e1002484.
21. Pillai MM, Gillen AE, Yamamoto TM, Kline E, Brown J, Flory K, Hesselberth JR, Kabos P. HITS-CLIP reveals key regulators of nuclear receptor signaling in breast cancer. Breast Cancer Res Treat. 2014;146:85-97.
22. Gottwein E, Corcoran DL, Mukherjee N, Skalsky RL, Hafner M, Nusbaum JD, Shamulailatpam P, Love CL, Dave SS, Tuschl T, Ohler U, Cullen BR. Viral microRNA targetome of KSHV-infected primary effusion lymphoma cell lines. Cell Host Microbe. 2011;10:515-26.
23. Cho H, Hwang M, Hong EH, Yu H, Park HH, Koh SH, Shin YU. Micro-RNAs in the aqueous humour of patients with diabetic macular oedema. Clin Exp Ophthalmol. 2020;48:624-635.
24. García-Martínez A, López-Muñoz B, Fajardo C, Cámara R, Lamas C, Silva-Ortega S, Aranda I, Picó A. Increased E2F1 mRNA and miR-17-5p Expression Is Correlated to Invasiveness and Proliferation of Pituitary Neuroendocrine Tumours. Diagnostics (Basel). 2020;10:227.
25. Yuan Y, Li X, Li M. Overexpression of miR 17 5p protects against high glucose induced endothelial cell injury by targeting E2F1 mediated suppression of autophagy and promotion of apoptosis. Int J Mol Med. 2018;42:1559-1568.
26. Zhang X, Song H, Qiao S, Liu J, Xing T, Yan X, Li H, Wang N. MiR-17-5p and miR-20a promote chicken cell proliferation at least in part by upregulation of c-Myc via MAP3K2 targeting. Sci Rep. 2017;7:15852.
27. Wu Q, Yang Z, Wang F, Hu S, Yang L, Shi Y, Fan D. MiR-19b/20a/92a regulates the self-renewal and proliferation of gastric cancer stem cells. J Cell Sci. 2013;126:4220-9.
28. O'Donnell KA, Wentzel EA, Zeller KI, Dang CV, Mendell JT. c-Myc-regulated microRNAs modulate E2F1 expression. Nature. 2005;435:839-43.
29. Trompeter HI, Abbad H, Iwaniuk KM, Hafner M, Renwick N, Tuschl T, Schira J, Müller HW, Wernet P. MicroRNAs MiR-17, MiR-20a, and MiR-106b act in concert to modulate E2F activity on cell cycle arrest during neuronal lineage differentiation of USSC. PLoS One. 2011;6:e16138.
30. Miao C, Yu H, Chang J, Zhang G, Zhou G, Zhao C. miR-148b-3p affects the pathogenesis of adjuvant-induced arthritis rats through the direct target DNMT1. Autoimmunity. 2018;51:43-52.
31. Tian L, Wu D, Dasgupta A, Chen KH, Mewburn J, Potus F, Lima PDA, Hong Z, Zhao YY, Hindmarch CCT, Kutty S, Provencher S, Bonnet S, Sutendra G, Archer SL. Epigenetic Metabolic Reprogramming of Right Ventricular Fibroblasts in Pulmonary Arterial Hypertension: A Pyruvate Dehydrogenase Kinase-Dependent Shift in Mitochondrial Metabolism Promotes Right Ventricular Fibrosis. Circ Res. 2020;126:1723-1745.
32. Chandra A, Sharma K, Pratap K, Singh V, Saini N. Inhibition of microRNA-128-3p attenuates hypercholesterolemia in mouse model. Life Sci. 2021;264:118633.
33. Yang Z, Wang J, Pan Z, Zhang Y. miR-143-3p regulates cell proliferation and apoptosis by targeting IGF1R and IGFBP5 and regulating the Ras/p38 MAPK signaling pathway in rheumatoid arthritis. Exp Ther Med. 2018;15:3781-3790.
34. Soriano-Arroquia A, McCormick R, Molloy AP, McArdle A, Goljanek-Whysall K. Age-related changes in miR-143-3p:Igfbp5 interactions affect muscle regeneration. Aging Cell. 2016;15:361-9.
35. Xihua L, Shengjie T, Weiwei G, Matro E, Tingting T, Lin L, Fang W, Jiaqiang Z, Fenping Z, Hong L. Circulating miR-143-3p inhibition protects against insulin resistance in Metabolic Syndrome via targeting of the insulin-like growth factor 2 receptor. Transl Res. 2019;205:33-43.
