# Supplementary figures and images for: EVOO Promotes a Less Atherogenic Profile Than Sunflower Oil in Smooth Muscle Cells Through the Extracellular Vesicles Secreted by Endothelial Cells
Source: Front Nutr. 2022 Apr 12;9:867745. doi: 10.3389/fnut.2022.867745 (PMC9039400; doi:10.3389/fnut.2022.867745)

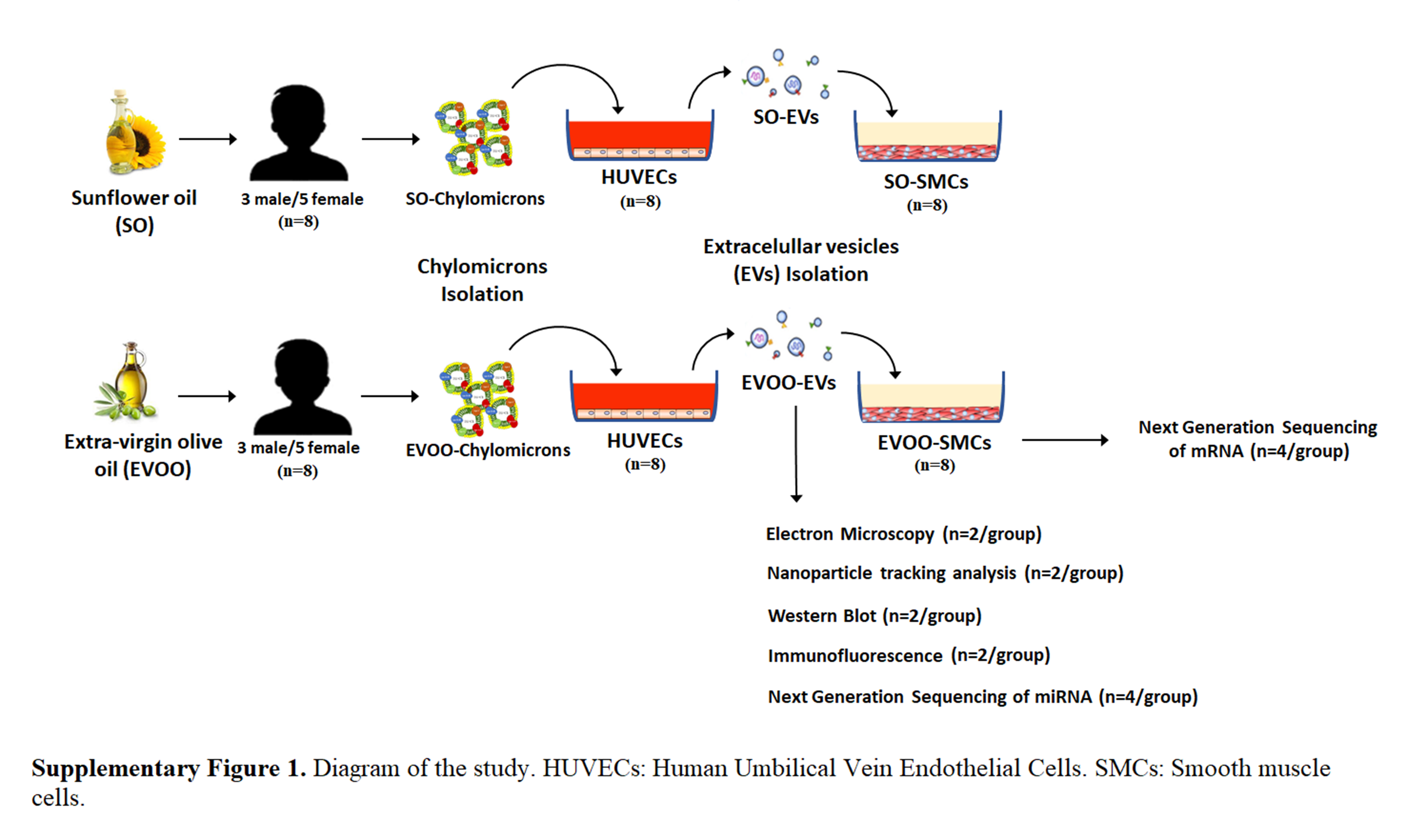

Supplement: Supplementary file 2 [file Image_1.TIF]
